# Supplementary material for: Tuning organic crystal chirality by the molar masses of tailored polymeric additives
Source: Nat Commun. 2021 Nov 25;12:6841. doi: 10.1038/s41467-021-27236-1 (PMC8617073; doi:10.1038/s41467-021-27236-1)
Supplement: Supplementary file 1 — Supplementary Information [file 41467_2021_27236_MOESM1_ESM.pdf]

Tuning organic crystal chirality by molar masses of tailored polymeric additives

Xichong Ye,[1] Bowen Li,[1] Zhaoxu Wang,[1] Jing Li,[1] Jie Zhang,[1] Xinhua Wan[1]*

[1] Beijing National Laboratory for Molecular Sciences, Key Laboratory of Polymer Chemistry and Physics of Ministry of Education, College of Chemistry and Molecular Engineering, Peking University, Beijing 100871, China.

Supplementary Information

■ Supplementary Notes

Supplementary Note 1. Materials: Methacryloyl chloride (97%, Energy Chemical), boc-4-amino-*L*-phenylalanine (98%, Energy Chemical), *N*-*α*-(*tert*-butoxycarbonyl)-*L*-lysine (98%, Energy Chemical), toluene-4-sulfonic acid monohydrate (*p*Ts•H_2O, 98%, Sinopharm Chemical Reagent Co.), *D*-4-hydroxyphenylglycine (*D*-*p*Hpg) and *L*-4-hydroxyphenylglycine (*L*-*p*Hpg) (98%, J&K Co.), (*2S,3R*)-threonine (*L*-Thr) and (*2R, 3S*)-threonine (*D*-Thr) (99%, TCI (Shanghai) Development Co.), (*2R,3R*)-threonine (*L*-aThr) and (*2S, 3S*)-threonine (*D*-aThr) (98%, Aladdin), *D*-aspartic acid (*D*-Asp) and *L*-aspartic acid (*L*-Asp) (99%, J&K Scientific), sodium sulfide (90%, J&K Scientific), cupric acetate monohydrate (99%, J&K Scientific), acetic acid (HPLC, Concord Technology (Tianjin) Co.), hydrochloric acid (36.0-38.0wt%, Xilong Scientific Co.), absolute ethyl ether (AR, Beijing Tongguang Fine Chemicals Co.) were used as purchased. Azobisisobutyronitrile (AIBN, AR, Sinopharm Chemical Reagent Co.) was recrystallized three times from ethanol and dried under vacuum at room temperature. Dioxane and tetrahydrofuran (AR, Beijing Chemical Co.) were refluxed with sodium and distilled before use. 4-Cyano-4-[(dodecylsulfanylthiocarbonyl)sulfanyl] pentanoic acid (CDP, 98%, Sigma-Aldrich) was recrystallized from hexane, and stored at 4 ºC. Fluorescein O-methacrylate (95%, Sigma-Aldrich) was purified by column chromatography (silica gel, dichloromethane/ethyl acetate (10/1, v/v) as eluent) and stored away from light.

Supplementary Note 2. Measurements: ^{1}H NMR experiments were carried out on a Bruker ARX400 spectrometer at room temperature using TMS as an internal standard. SEM images were obtained on a Hitachi S-4800 field emission scanning electron microscope operated at 10 KV. The samples in early stage of crystallization were prepared by dipping a drop of solution onto a hydrophilic cleaning silica base keeping at 25 °C and the solutions were absorbed by dust-free paper after 30 s. the samples in later stage of crystallization were prepared by filtration, and the crystals were stick to conductive adhesive. The number-average molar masses (M_{n}), weight-average molar masses (M_{w}), and polydispersity indices (PDI=M_{w}/M_{n}) of the resultant polymers were estimated on a gel permeation chromatographic (GPC) instrument equipped with a Waters 515 HPLC pump and a Waters 2410 refractive-index detector. Three Waters Styragel columns with 10 mm bead size were connected in tandem. Their effective molar masses ranges were 100-10000 for Styragel HT2, 500-30000 for Styragel HT3, and 5000-600000 for Styragel HT4, respectively. The pore sizes were 50, 100, and 1000 nm for Styragels HT2, HT3, and HT4, respectively. THF was used as the eluent at a flow rate of 1.0 mL min^{-1} at 35 °C. The calibration curve was obtained against polystyrene standards. A J-810 circular dichroism spectrometer (Jasco Corporation, Japan) was involved to achieve the circular dichroism spectra. Fluorescence (FL) spectra were obtained on Hitachi F7000 spectrometer, the excitation wavelength was 435.0 nm, the emission was recorded from 400 nm ~ 700 nm, the scan speed was 60 nm/min, the excitation slit was 10.0 nm, the emission slit was 5.0 nm and the PMT voltage was 600 V. 2D and 3D Fluorescence images were obtained using a laser scanning confocal microscope Nikon A1R-si, the excitation wavelength was 405 nm. Dynamic light scatter (DLS) measurements were performed on a commercialized spectrometer from Brookhaven Instrument Corporation (BI-200SM Goniometer, Holtsville, NY). A vertically polarized, 100 mW solid-state laser (GXC-III, CNI, Changchun, China) operating at 633 nm was used as the light source, and a digital correlator (Brookhaven Instruments Corp.) was used to collect and process data. The samples were filtered through 220 nm filters. The single crystal of *D*- and *L*-*p*Hpg*p*Ts were measured using an X-ray single crystal diffractometer (XtaLAB PRO 007HF, Rigaku, Japan) with MoKα (λ = 0.71 Å) radiation. Metastable zones were measured in a commercially available reactor system (EasyMax 102, Mettler Toledo, Switzerland) equipped with the probes for in-situ monitoring of the crystals formation, dissolution and particle vision (ParticleTrack G600B).

The ee% was estimated on a high-performance liquid chromatography (HPLC) equipped with a JASCO PU-2089 pump, a AS-2055 automatic sampler, a UV-2070 UV-Vis spectrometer, a CD-2095 circular dichroism spectrometer and a Daicel CROWNPAK CR(+) column. Perchloric acid aqueous solution was used as the eluent (*p*H = 1; flow rate, 0.8 mL min^{-1}; temperature, 37 ºC).

■ Supplementary Methods:

Synthesis of *p*-methacrylamido-*α*-*tert*-butoxycarbonyl-*L*-phenylalanine. (*L*-MPABoc). Boc-4-amino-*L*-phenylalanine (5.0 g, 17.8 mmol) was dissolved in 65 mL of water. The solution of the methacryloyl chloride (2.2 g, 21.1 mmol) in 40 mL of dry THF was added dropwise into the solution at 0 ºC with vigorous stirring, and keeping the pH value close to 9 by gradual addition of 1 M NaOH aqueous solution. Keep stirring at room temperature for 1 day, followed by washing with 3 × 150 mL portions of diethyl ether. The aqueous solution was acidified with 0.5 M HCl until pH close to 3. Then the mixture was extracted with 3 × 150 mL portions of ethyl acetate. The organic layers were combined, and dried over anhydrous Na_2SO_4. After evaporation of the solvent under reduced pressure, the crude product was purified further by column chromatography (silica gel, dichloromethane/methanol (20/1, v/v) as eluent) and recrystallized in CH_2Cl_2 to give 4.9 g of white solids. Yield: 78 %.

^{1}H NMR (400 MHz, DMSO-d_6, δ, ppm): 1.33 (s, 9H; -C$(CH_3)_3$), 1.94 (s, 1H; =C(CH_3)-), 2.78-2.95 (m, 2H; Ar-CH_2-), 4.04 (m, 1H; -CH_2-*CH*(COOH)-), 5.49-5.77 (d, 2H; vinyl), 7.07 (s, 1H; NH), 7.18-7.56 (m, 4H; Ar-H), 9.71 (s, 1H; NH), 12.58 (s, 1H; -COOH).

Synthesis of poly[*p*-methacrylamido *tert*-butoxycarbonyl-*L*-phenylalanine] (*L*-PMPABoc). A typical process for the polymerization was presented as follows: *L*-MPABoc (3.5 g, 10.0 mmol), AIBN (3.3 mg, 0.020 mmol), CDP (40.4 mg, 0.10 mmol), and dioxane (35.0 g) (molar ratio of MPABoc/CDP/AIBN = 500/5/1) were introduced into a polymerization tube with a magnetic bar. After three freeze-pump-thaw cycles, the tube was flame-sealed under vacuum and put into an oil-bath thermostatted at 80 ºC. After the polymerization continued for 42 h, the tube was cooled to room temperature and broken. The solution was diluted with 30 mL of THF and added dropwise into 800 mL of diethyl ether. The precipitated solids were collected by filtration and dried under vacuum for 24 h at room temperature to give 2.84 g of yellow powders. Yield:

81%. After methyl esterification, the polymer is tested by GPC, the M_n is 15703, and the PDI is 1.28. The obtained polymers were further purified by preparative GPC to obtain narrowly distributed polymers (PDI~1.10)

^{1}H NMR (400 MHz, DMSO-d_6, δ, ppm): 0.4-1.5 (-C(CH_3), -CH_3, main chain CH_2), 2.5-3.3 (CH_2 and CH in phenylalanine), 6.4-6.8 (NH), 6.8-7.7 (Ar-H), 8.6-9.4 (NH) and 12.4-12.7 (COOH).

Synthesis of fluorescein-labeled *L*-PMPABoc[1]. MPABoc (200 mg, 574 μmol), Fluorescein O-methacrylate (400.38 mg, 17.2 μmol), AIBN (0.2 mg, 1.22 μmol), CDP (2.32 mg, 5.75 μmol), and dioxane (2.0 g) (molar ratio of MPABoc/ Fluorescein O-methacrylate/CDP/AIBN = 500/20/5/1) were introduced into a polymerization tube with a magnetic bar. After three freeze-pump-thaw cycles, the tube was flame-sealed under vacuum and put into an oil-bath thermostatted at 80 ºC. After the polymerization continued for 42 h, the tube was cooled to room temperature and broken. The solution was diluted with 3 mL of THF and added dropwise into 80 mL of diethyl ether. The precipitated solids were collected by filtration and dried under vacuum for 24 h at room temperature to give 150 mg of yellow powders. Yield: 76%. After methyl esterification, the polymer is tested by GPC, the Mn is 14363, and the PDI is 1.26.

Synthesis of N^2-(*tert*-butoxycarbonyl)-N^6-methacryloyl-*L*-lysine (*L*-MALBoc). *α*-(*tert*-Butoxycarbonyl)-*L*-lysine (10.0 g, 40.6 mmol) was dissolved in 70 mL of water. The solution of the methacryloyl chloride (5.1 g, 48.8 mmol) in 48 mL of dry THF was added dropwise into the *α*-(*tert*-butoxycarbonyl)-*L*-lysine solution at 0 ºC with vigorous stirring, and keeping the pH value close to 9 by gradual addition of 1 M NaOH aqueous solution. Keep stirring at room temperature for 1 day, followed by washing with 3 × 150 mL portions of diethyl ether. The aqueous solution was acidified with 0.5 M HCl until pH close to 3. Then the mixture was extracted with 3 × 150 mL portions of ethyl acetate. The organic layers were combined, and dried over anhydrous Na_2SO_4. After evaporation of the solvent under reduced pressure, the crude product was purified further by column chromatography (silica gel, dichloromethane/methanol (20/1, v/v) as eluent) and recrystallized in CH_2Cl_2 to give 10.2 g of white solids. Yield: 80 %.

^{1}H NMR (400 MHz, CDCl3, δ, ppm): 1.44 (m, 11H; $-C(\mathit{CH_3})_3$ & $-CH_2CH_2\mathit{CH_2}CH_2CH-$), 1.55-1.62 (m, 2H; $-CH_2\mathit{CH_2}CH_2CH_2CH-$), 1.70-1.87 (m, 2H; $-CHCH_2CH_2\mathit{CH_2}CH-$), 1.95 (s, 1H; $=C(\mathit{CH_3})-$), 3.29-3.34 (m, 2H; $-\mathit{CH}CH_2CH_2CH_2CH-$), 4.12-4.29 (m, 1H; $-CHCH_2CH_2CH_2\mathit{CH}-$), 5.30-5.35 (m, 2H; vinyl & NH), 5.70 (s, 1H; vinyl), 6.13-6.23 (m, 1H; NH), 9.94 (broad, 1H; -COOH).

Synthesis of Poly[N^2-(*tert*-butoxycarbonyl)-N^6-methacryloyl-*L*-lysine] (*L*-PMAlBoc). A typical process for the polymerization was presented as follows: *L*-MALBoc (0.95 g, 3.0 mmol), AIBN (0.98mg, 0.0060 mmol), CDP (24.2mg, 0.060 mmol), and dioxane (6.0 g) (molar ratio of *L*-MALBoc/CDP/AIBN = 500/10/1) were introduced into a polymerization tube with a magnetic bar. After three freeze-pump-thaw cycles, the tube was flame-sealed under vacuum and put into an oil-bath thermostatted at 65 ºC. After the polymerization continued for 10 h, the tube was cooled to room temperature and broken. The solution was diluted with 10 mL of THF and added dropwise into 150 mL of diethyl ether. The precipitated solids were collected by filtration and dried under vacuum for 24 h at room temperature to give 0.45 g of yellow powders. Yield: 48%.

^{1}H NMR (400 MHz, DMSO-d_6, δ, ppm): 0.7-1.6 ($-C(CH_3)$, $-CH_3$, main chain CH_2, and CH_2 in Lys), 2.9-3.5 (CH_2 in Lys), 3.7-3.9 (CH in Lys), 6.6-7.2 (NH), and 12.4 (COOH). FTIR (neat, KBr plate, wavenumber, cm-1): 3393, 2978, 2936, 2869, 1711, 1646, 1527, 1457, 1394, 1367, 1251, 1168.

Deprotection of N-Boc group (*L*-PMPA•HCl or *L*-PMAL•HCl). Polymer (500 mg) and THF (30 mL) was added into a 50 mL flask, and keep stirring for 1 h until the solution become homogeneous. Hydrochloric acid (10 M, 3 mL) was added dropwise into this solution, and then the mixture was stirred for 6 h at room temperature, and a gelatinous sediment appeared. After pouring the solvent out, 50 mL diethyl ether was added to the crude product and stirred for 1 h. Pouring the solvent out and repeat this process for 5 times. The precipitated solids were filtered and then dried under vacuum at 35 ºC for 12 h. Yield: ~100%.

Metastable zone test. The metastable zone of *rac*-*p*Hpg*p*Ts solution in the presence or absence of P5 were measured (Supplementary Fig. 3). Samples with different concentration of *rac*-*p*Hpg*p*Ts were prepared, the clearing points when heating and the cloud points when cooling were recorded to draw the curves.

Selective crystallization of *rac*-*p*Hpg*p*Ts. In a typical process by using *rac*-*p*Hpg*p*Ts as substrates and *L*-PMPAHCl as additives, the supersaturated solution (2.5 g of *rac*-*p*Hpg*p*Ts in 10 mL 0.5 M *p*-Ts solution) was heated at 60 ºC until complete dissolution occurred, filtered, and then 2.5 g supersaturated solution was transferred to a hot penicillin bottle, a certain amount of additives' solution (200 mg•mL^{-1} in water) was added in. The whole solution was gradually cooled down to 25 ºC (2 ºC per 10 min). After being left stand at 25 ºC for 10 min, seeds of *D*-*p*Hpg*p*Ts were added in. After a period of time, the supernatant fluid was collected and the formed crystals were washed with cold acetone, and then dried under vacuum.

Cooling crystallization from a diluted solution was conducted for the CD test to prevent the HT voltage from exceeding the threshold. Besides, the absorption of *p*Hpg*p*Ts is around 240 nm and the CD signal is weak and unreliable. In order to solve this problem, we do the crystallization by adding disodium sulphonated bathophenanthroline (DSB) to replace 5 mol% of the *p*-toluenesulfonic acid. The obtained red crystals were used to test the CD signals. The DSB were used as probe to detect the chirality.

Calculation methods.

Crystal habit in vacuum. All the calculation and simulations were carried out in Materials Studio 2017. The unit cell of *D*/*L*-*p*Hpg*p*Ts was optimized by the Drieding force field. The crystal habit of *D*/*L*-*p*Hpg*p*Ts in vacuum was generated through AE model by using the morphology module. And a table containing E_{att}(hkl), *d*(hkl), total facet area (%) can also be obtained (Supplementary Table 3,4). The prediction of the crystal habit in vacuum resulted in a prismatic morphology (Supplementary Fig. 18). The exhibiting facet families are {0 1 1}, {0 0 2}, {1 1 0}, and {1 0 1}.

Crystal habit in pure water. The *D*/*L*-*p*Hpg*p*Ts crystal is cleaved parallel to the (0 1 1), (0 0 2), (1 1 0), and (1 0 1) plane with a depth of 2×*d*(*hkl*). A crystal layer is constructed as a periodic superstructure of 3 × 3 unit cells. Solvent layers, which sizes were set consistent with the crystal layers, were constructed using the Amorphous Cell module with 200 randomly distributed water molecules and refined by geometry optimization. Two-layered interfacial model was built with a crystal layer and a corresponding solvent layer, the solvent layer is adsorbed on the (h k l) crystal face along c axis. And a vacuum of 50 Å thickness was placed onto the solvent layer to eliminate the effect of additional free boundaries on the structure. In order to study the effect of solvent during the process of crystal growth, MD simulation with an NVT ensemble at 298 K (100 ps with a time step of 1 fs, controlled by an Andersen thermostat) was employed. The Coulombic and van der Waals interactions are calculated by employing the atom-based method with a cut-off distance of 30 Å. The crystal slice is constrained during the MD simulation process. All the calculations are based on the Drieding force field. Tables containing E_{att}(hkl)', R' and total facet area (%) can be obtained (Supplementary Table 5,7)

Crystal habit in *L*-monomer's solution. Solution models have been constructed with 10 *L*-monomers and 190 water molecules (5%) or 15 *L*-monomers and 185 water molecules (7.5%) which are randomly distributed in the box. And the operations are the same as the previous one. Tables containing E_{att}(hkl)', R' and total facet area (%) can be obtained (Supplementary Table 6, 8 and 9)

Calculation of the adsorption angle. The adsorption angle θ is the growth angle between the main crystals and attached crystals. It is known that the (0 1 1) face is the preferred surface when the attachment of small crystals happens. Therefore, The *D*/*L*-*p*Hpg*p*Ts crystal is cleaved parallel to the (0 1 1) plane with a depth of 2×*d*(*hkl*). A main crystal layer is constructed as a periodic supercell of 18 × 18 unit cells and an attached crystal layer is constructed as a periodic supercell of 3× 3 unit cells. Two-layer interfacial model was built with a main crystal layer and an attached crystal layer along the c axis. The upper layer of the model was rotated from -6 to +6 degrees to simulate the real angle. Geometry optimization by the Drieding force field was conducted to

calculate the total energy of the model system. Then, the connection between the total energy E and the adsorption angle θ was plotted.

■ Supplementary theory:

Attachment energy model (AE model). AE model was proposed by Hartman and Bennema in 1980s[2,3]. It has been proved that the lower the absolute attachment energy of a facet family {h k l}, the slower the growth of the facet. The attachment energy (E_{att}), which indicates the energy released when a slice was added onto the {h k l} plane from an infinite distance, is proportional with the relative growth rates ($R(hkl)$) and the distance of the crystal surface to the centre ($D(hkl)$) (1). This model is usually used to predict the crystal habit in vacuum. The AE model has been included in the package of Modules in Materials Studio 2017.

$$D(hkl) \propto R(hkl) \propto |E_{att}(hkl)| \quad (1)$$

Corrected attachment energy model[4-7]. The solvents and additives can significantly influence the crystal habit. These molecules have to be removed from the surface before the crystal face can continue to grow. This costs energy and decreases the attachment energy. Thus, an energy correction term E_s for E_{att} must be introduced to generate the crystal habit in solutions (2):

$$E'_{att}(hkl) = E_{att}(hkl) - E_s(hkl) \quad (2)$$

The E_{att}'(hkl) represents the solvent-involved attached energy of a {h k l} slice. The more the absolute attachment energy of a {h k l} slice decreased, and the greater morphology importance (MI) would be obtained. Since the effect of solvents or additives depends on both the exposed functional groups and the surface roughness of a specific crystal face, the correction term E_s can be calculated as:

$$E_s(hkl) = E_{int}(hkl) \times S = \frac{E_{int}(hkl) \times A_{acc}(hkl)}{N \times A_{model}(hkl)} \quad (3)$$

E_{int} is the interaction energy between the solvent and the surface, $A_{model}(hkl)$ is the surface area of the simulated model in the {h k l} direction, $A_{acc}(hkl)$ is the accessible solvent surface area of the simulated model in the {h k l} direction. It can be obtained by calculating the Connolly surface. As the correction term E_s is to modify E_{att}, which is calculated based on a single unit cell, the whole term must be divided by N (number of repeat units in the simulated model). The E_{int} can be calculated as:

$$E_{int}(hkl) = -E_b(hkl) = E_{tot}(hkl) - E_{sur}(hkl) - E_{sol}(hkl) \quad (4)$$

$E_{tot}(hkl)$ is the total energy of the surface and the solvent after dynamic simulation, $E_{sur}(hkl)$ is the energy of the bare surface, and $E_{sol}(hkl)$ is the energy of the solvent layer without the surface. The binding energy ($E_b(hkl)$) is defined as the negative value of the interaction energy. The relative growth rate $R(hkl)$ for each face is proportional to $E'_{att}(hkl)$ (5), thus, face with the lowest absolute attachment energy should grow the slowest and have the highest morphological importance.

$$D(hkl) \propto R(hkl) \propto |E'_{att}(hkl)| \quad (5)$$

■ **Supplementary Figures**

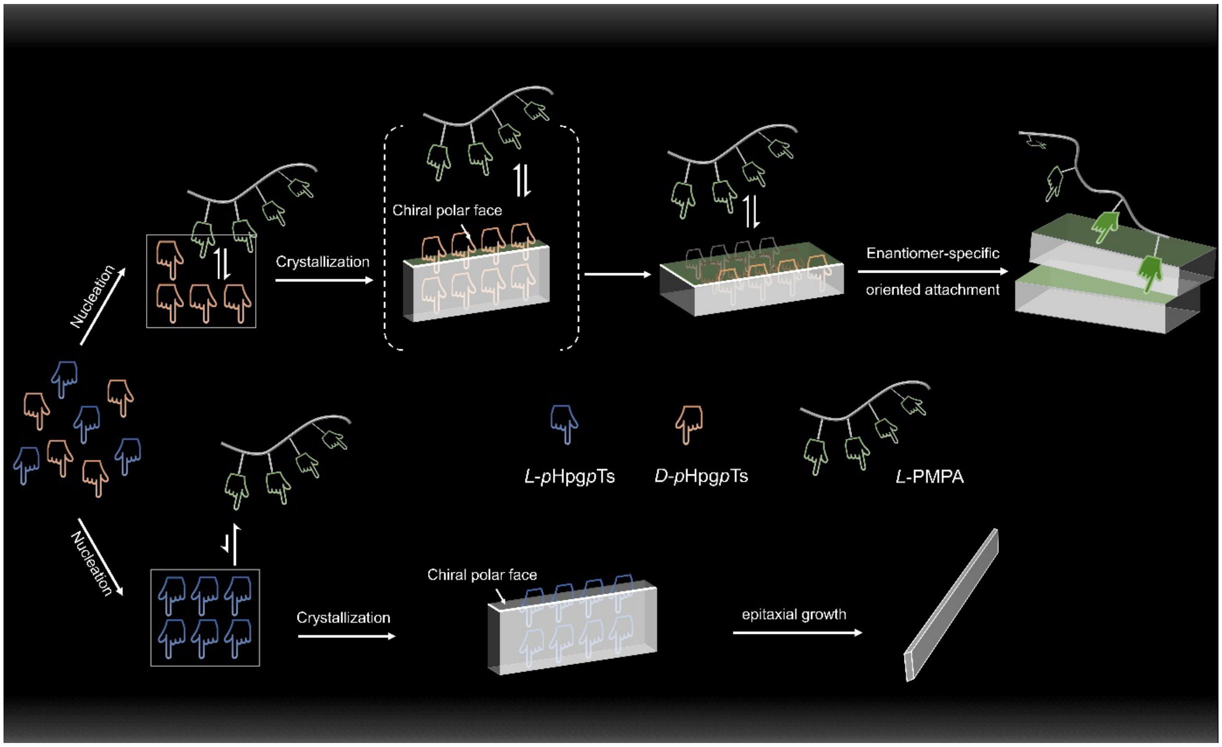

Supplementary Figure 1. The scheme of our strategy when short chain polymers were used as additive: *L*-PMPA modify the crystal habit of *L*-*p*Hpg*p*Ts through stereoselective interactions, while the interaction between *L*-PMPA and *D*-*p*Hpg*p*Ts is too weak to make any effluence. Thus, a mixture of M-type fan-shaped *L*-crystals and needle-like *D*-crystals were obtained.

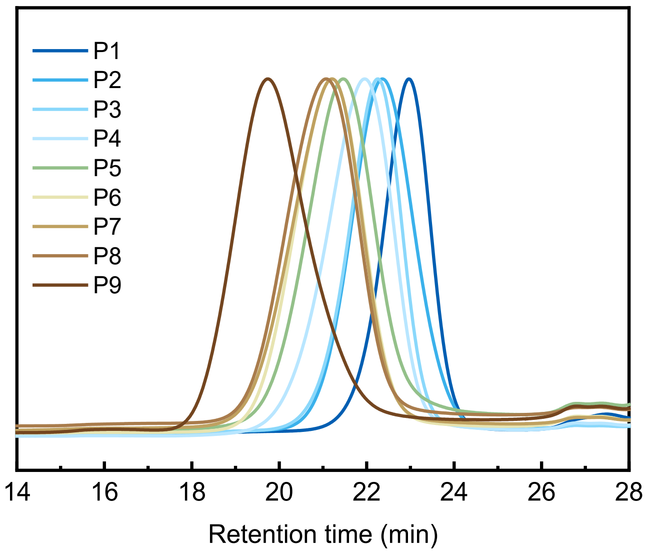

Supplementary Figure 2. the GPC curves of *L*-PMPABocOCH_3 (P1-P9) with various molar masses.

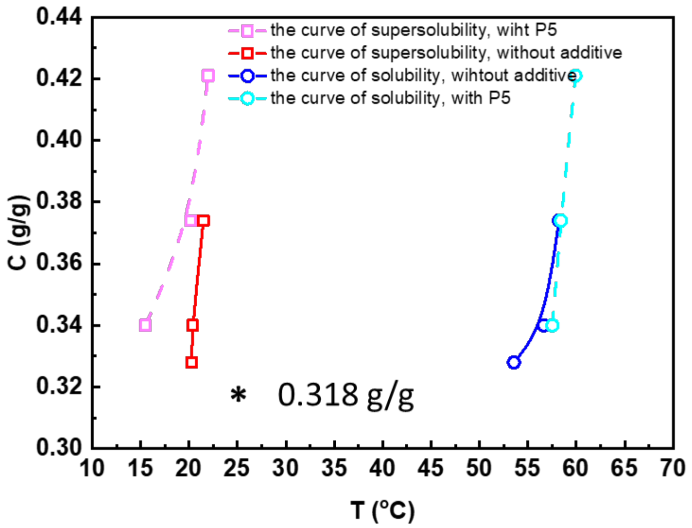

Supplementary Figure 3. The supersolubility and solubility curves of *rac*-*p*Hpg*p*Ts with (dashed lines) or without (solid lines) additives (P5). It is found that the metastable zone was broader when the polymers were added in, indicating the delay of nucleation caused by the polymers. The crystallization of *rac*-*p*Hpg*p*Ts was carried out within the metastable zone to avoid massive undesired spontaneous nucleation.

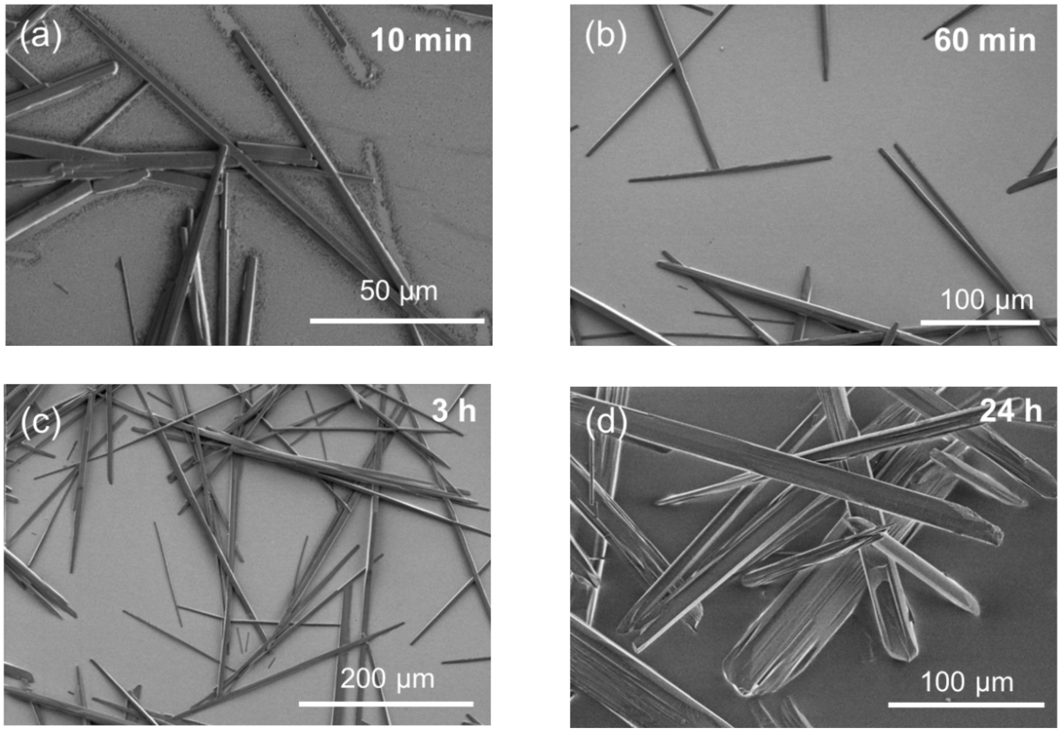

Supplementary Figure 4. The SEM images of crystals obtained at different time when no additives were used. **a** Crystals obtained at 10 min. **b** Crystals obtained at 60 min. **c** Crystals obtained at 3 h. **d** Crystals obtained at 24 h.

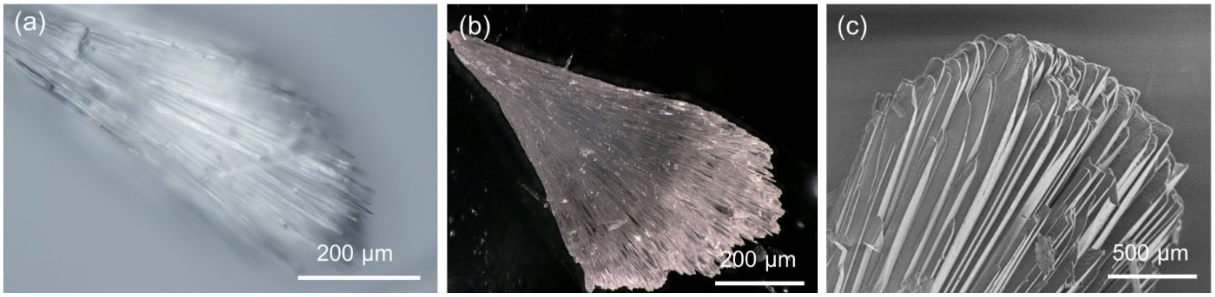

Supplementary Figure 5. Morphologies of P-type fan-shaped crystal aggregates of *D*-*p*Hpg*p*Ts. **a** Optical micrograph of one fan-shaped crystal. **b** Image taken by optical microscope with a large depth-of-field. **c** SEM image of an enlarged fan-shaped crystal.

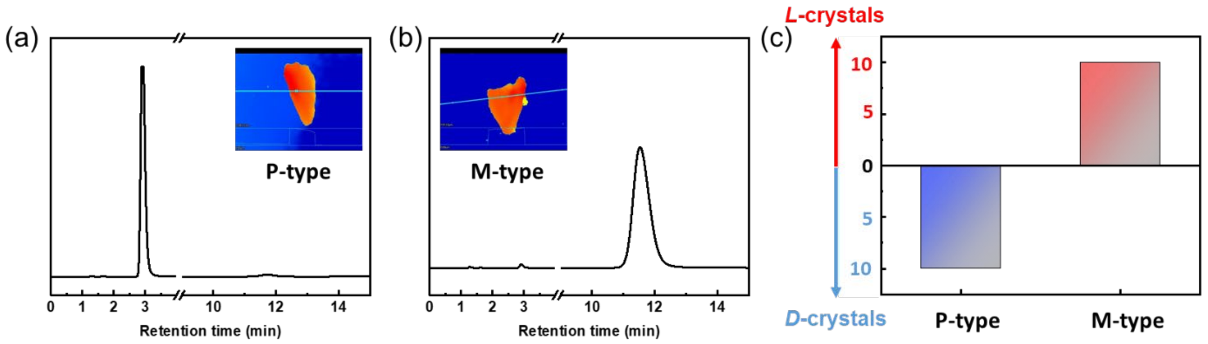

Supplementary Figure 6. Chiral HPLC results of the fan-shaped crystals. **a** Typical HPLC result of a P-type fan-shaped crystal. **b** Typical HPLC result of a M-type fan-shaped crystal. **c** Statistics of fan-shaped crystals configurations.

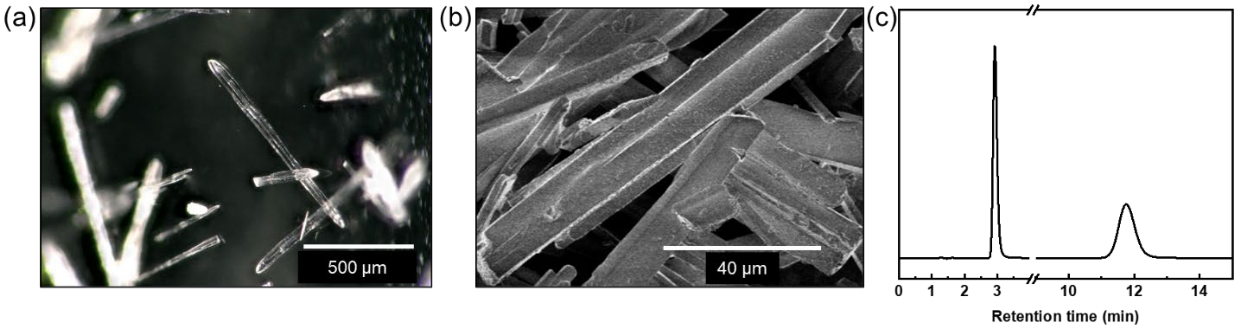

Supplementary Figure 7. The images of needle-like crystals obtained from *rac*-*p*Hpg*p*Ts solution without additives and their ee% values. **a** Image obtained by optical microscope with a large depth-of-field. **b** Image obtained by SEM. **c** Chiral HPLC result, the peak at 3 min represents *D*-*p*Hpg, the peak at 4.8 min represents *p*Ts (omitted in this picture) and the peak at 12 min represents *L*-*p*Hpg. The ee% values were calculated to be 0.9 % in this case.

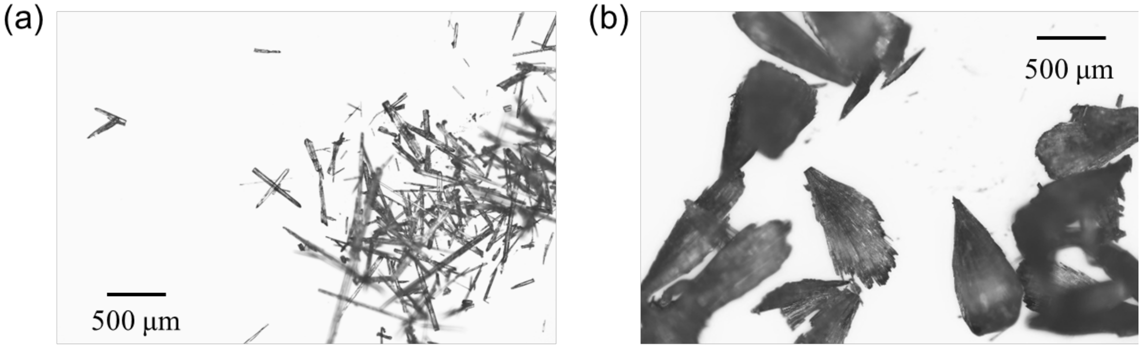

Supplementary Figure 8. The crystals obtained from *L*-*p*Hpg*p*Ts solution. **a** No additives were used. **b** 1.5 $_{wt}$% P5 were used as additive.

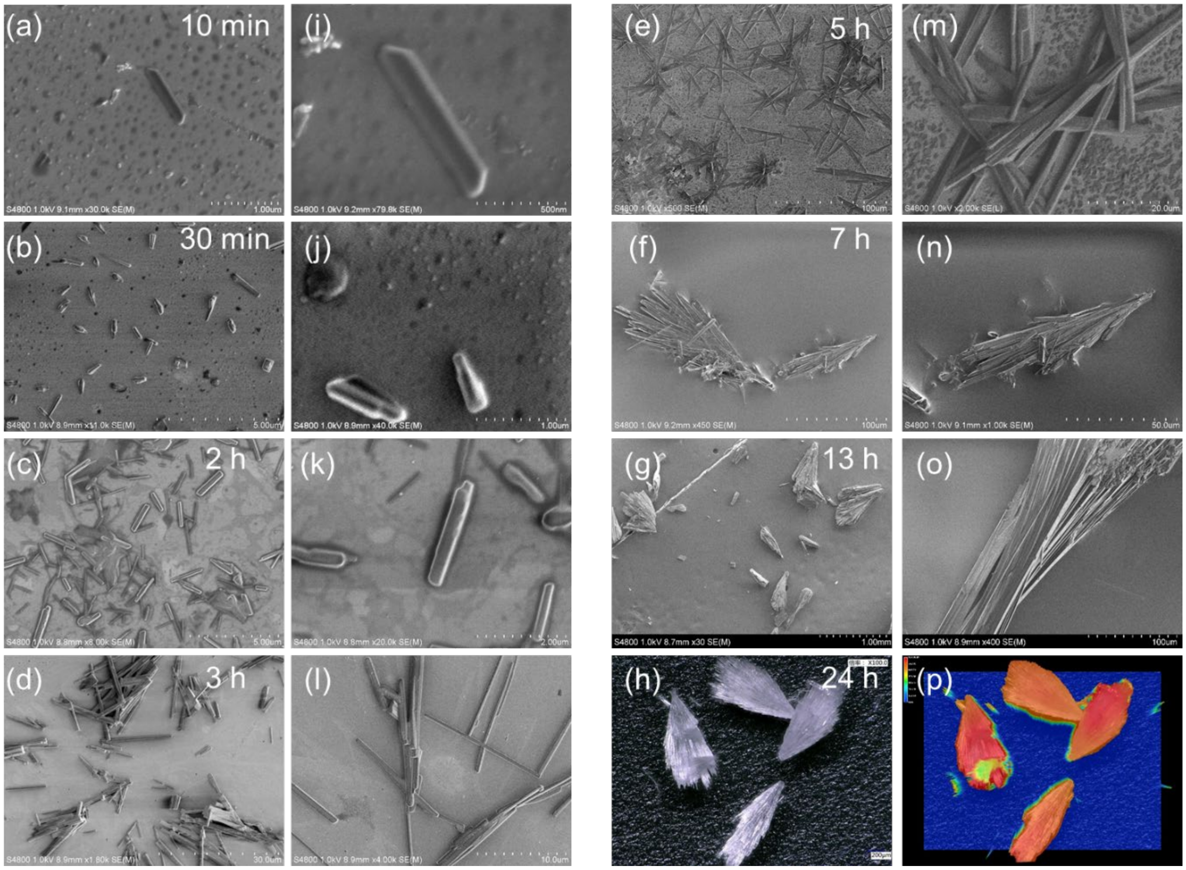

Supplementary Figure 9. The images of crystals obtained at different time when P5 was used as additive. **a-g** The SEM images of crystals obtained from 10 min to 24 h. **i-o** The enlarged SEM images corresponding to the left pictures. **h** Images obtained by optical microscope with a large depth-of-field at 24 h. **p** The height map of **h**.

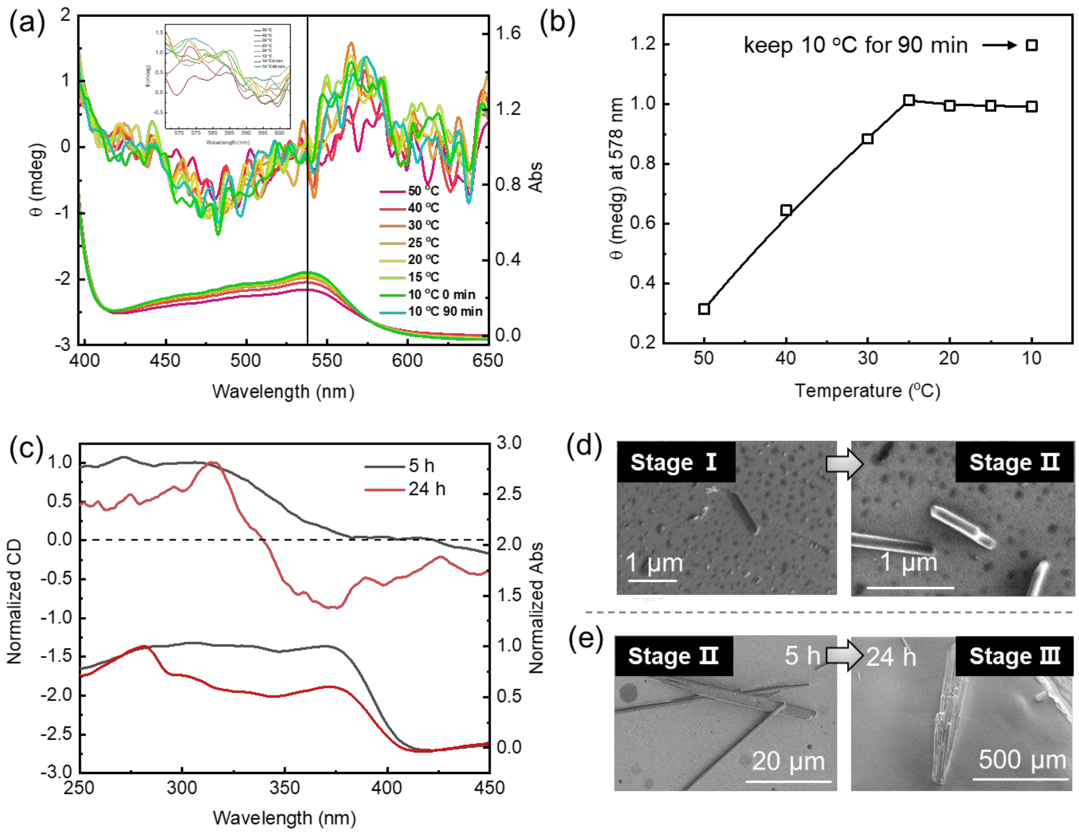

Supplementary Figure 10. The evolution of P-type fan-shaped crystals recorded by circular dichroic (CD) spectra. Disodium sulphonated bathophenanthroline (DSB) was used as probe to detect their chirality (see details in supplementary methods). A diluted solution was prepared, and the temperature was gradually dropped down to trigger the crystallization. 1.5 wt% P5 was used as the additive to generate fan-shaped *D*-crystals. The evolution between stage I and II was recorded on the transmission mode, as the whole solution is slightly turbid and no obvious precipitate can be observed. **a** It displayed two opposite bisignated signals in the visible region, a negative signal at 478 nm and a positive one at 578 nm. **b** The correlation between CD signal at 578 nm and temperature. From 50 to 25 °C, the CD signals gradually increased, which indicated the formation of primary nano-scaled building blocks. From 25 to 10 °C, no complex structures generated and the CD signal kept steady. When the sample was kept at 10 °C for 90 min, the initial assembly of two or three needle-like crystals occurred and the CD signals increased again. After 90 min, the crystals precipitated and the transmission mode cannot be used. The crystals generated at 5 h and 24h were collected to test their CD signals on diffuse mode. It is worth noting that there is no comparability between the CD signals of solutions and solids. **c** The crystals obtained at 5 h have a broad positive signal below 350 nm, while the crystals obtained at 24 h showed obvious cotton effect at the same wavelength. The cotton effect indicated the emergence of high-level chirality and better arrangement of chromophores.

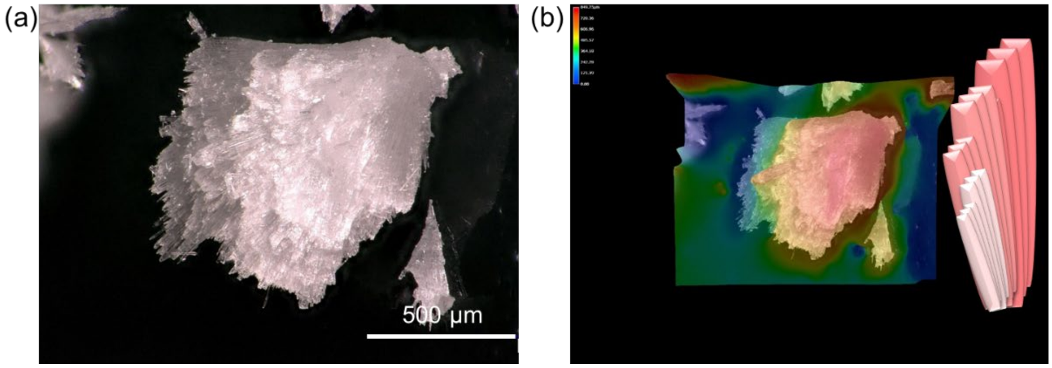

Supplementary Figure 11. a Typical image of a fan-shaped *D*-crystals composed of several fan-shaped sub-units. **b** The corresponding height map and a cartoon for the possible structure.

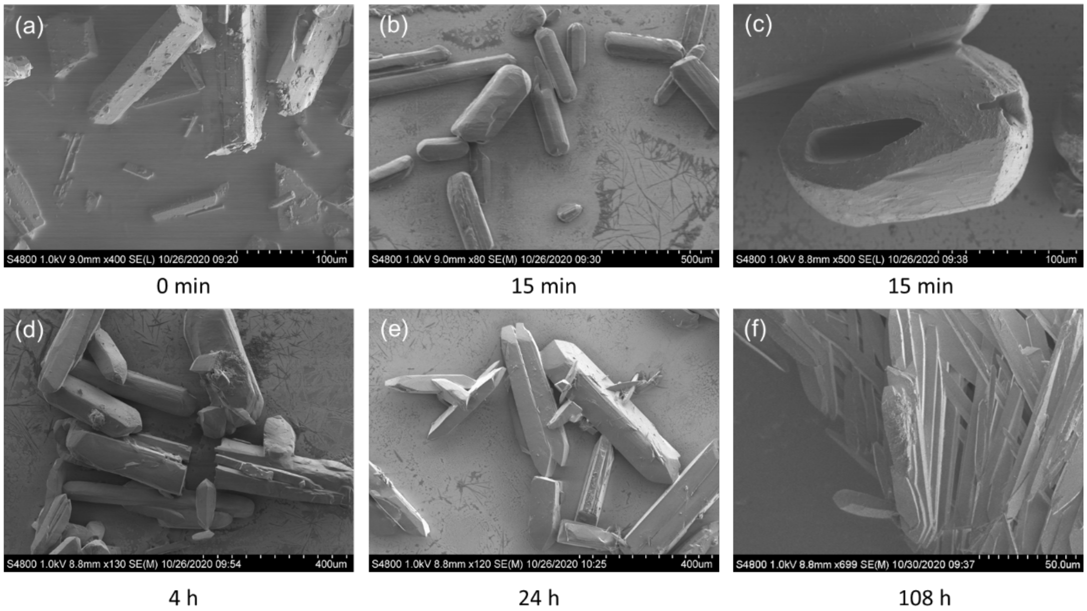

Supplementary Figure 12. Morphological changes of pre-prepared *D*-crystals in the saturated solution at different time. **a** 0 min. **b** 15 min. **c** Enlarged picture of one crystal at 15 min. **d** 4 h. **e** 24 h, **f** 108 h.

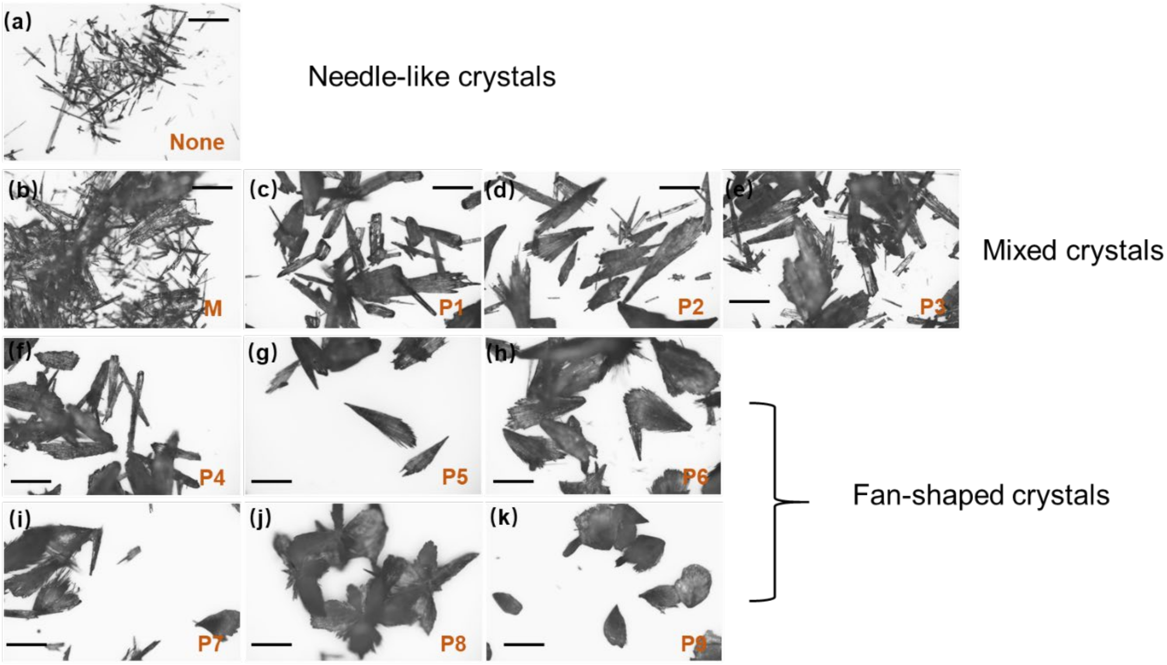

Supplementary Figure 13. The obtained crystals' morphologies by using different additives. **a** No additives. **b** *L*-monomers were used as additives. **c-k** P1-P9 were used as additives.

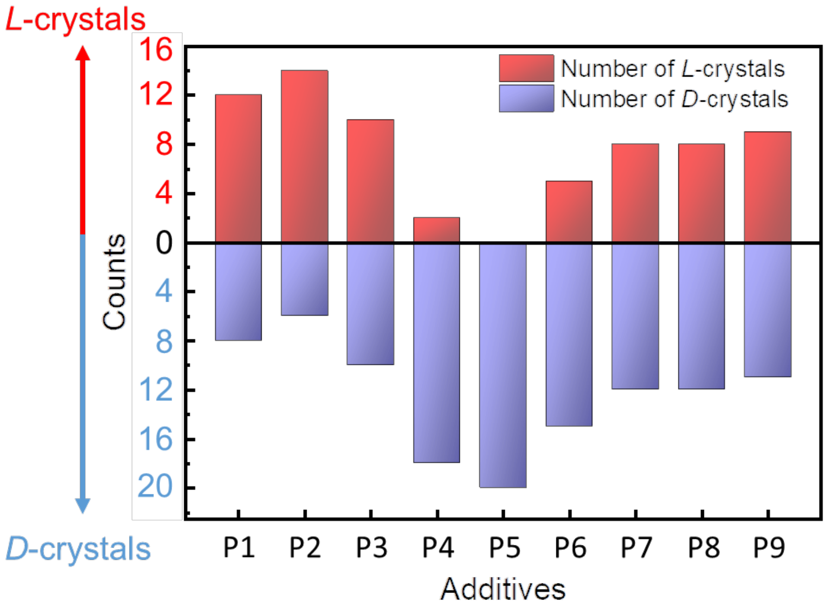

Supplementary Figure 14. Statistics of fan-shaped crystals configuration when polymers with various molar masses were used as additives.

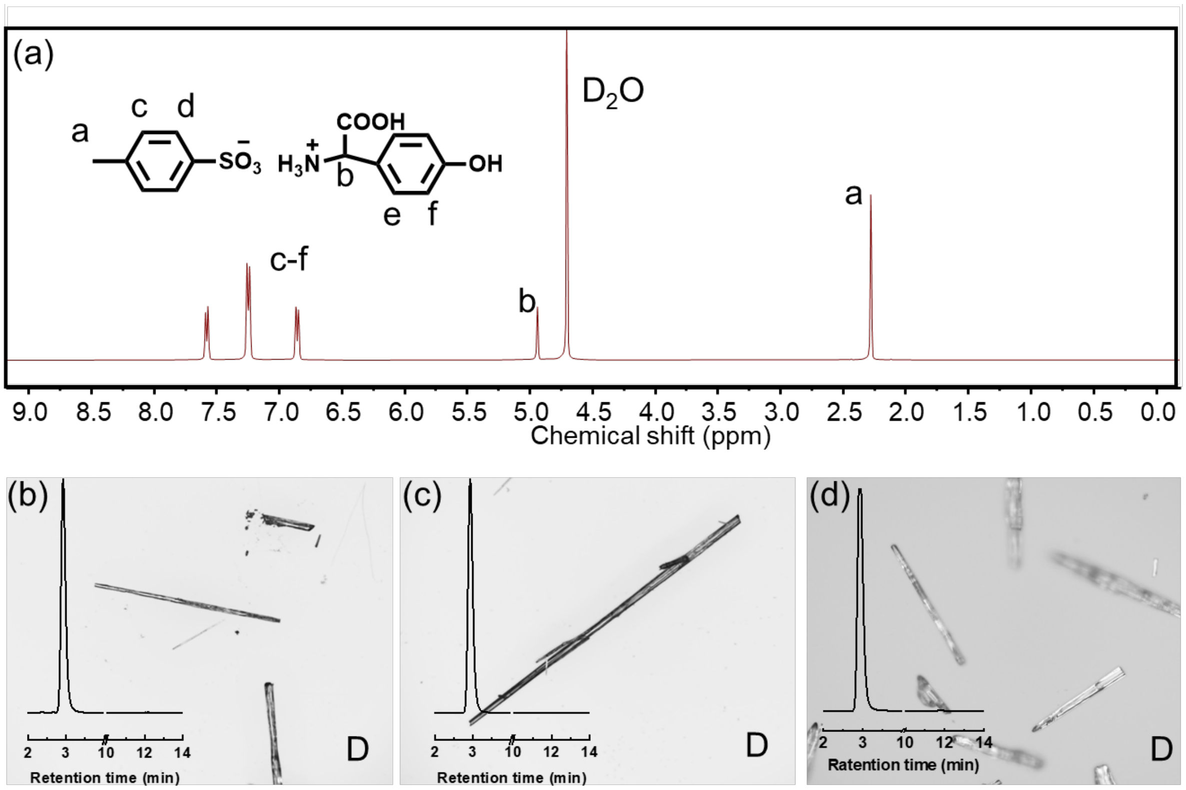

Supplementary Figure 15. The characterization of needle-like crystals obtained by using P1-P3 as additives. **a** The ^{1}H-NMR of needle-like crystals. **b-d** The images of the picked-out crystals and the chiral HPLC result.

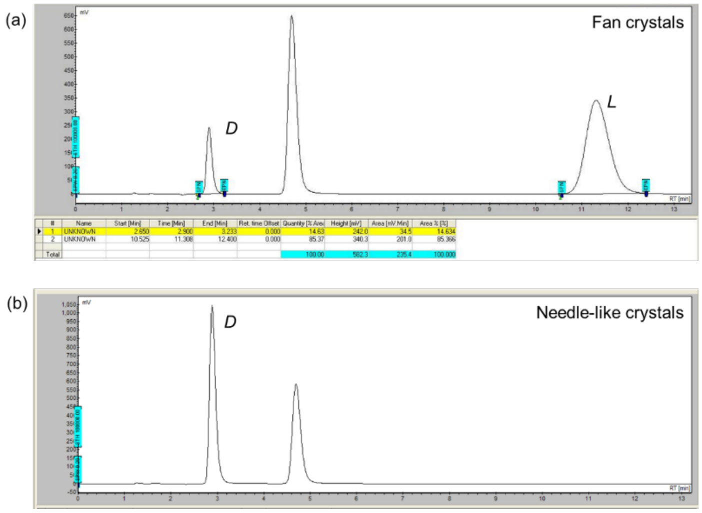

Supplementary Figure 16. The HPLC results of the *p*Hpg*p*Ts crystals obtained by using P2 as additive. (a) The separated fan-shaped crystals. (b) The separated needle-like crystals.

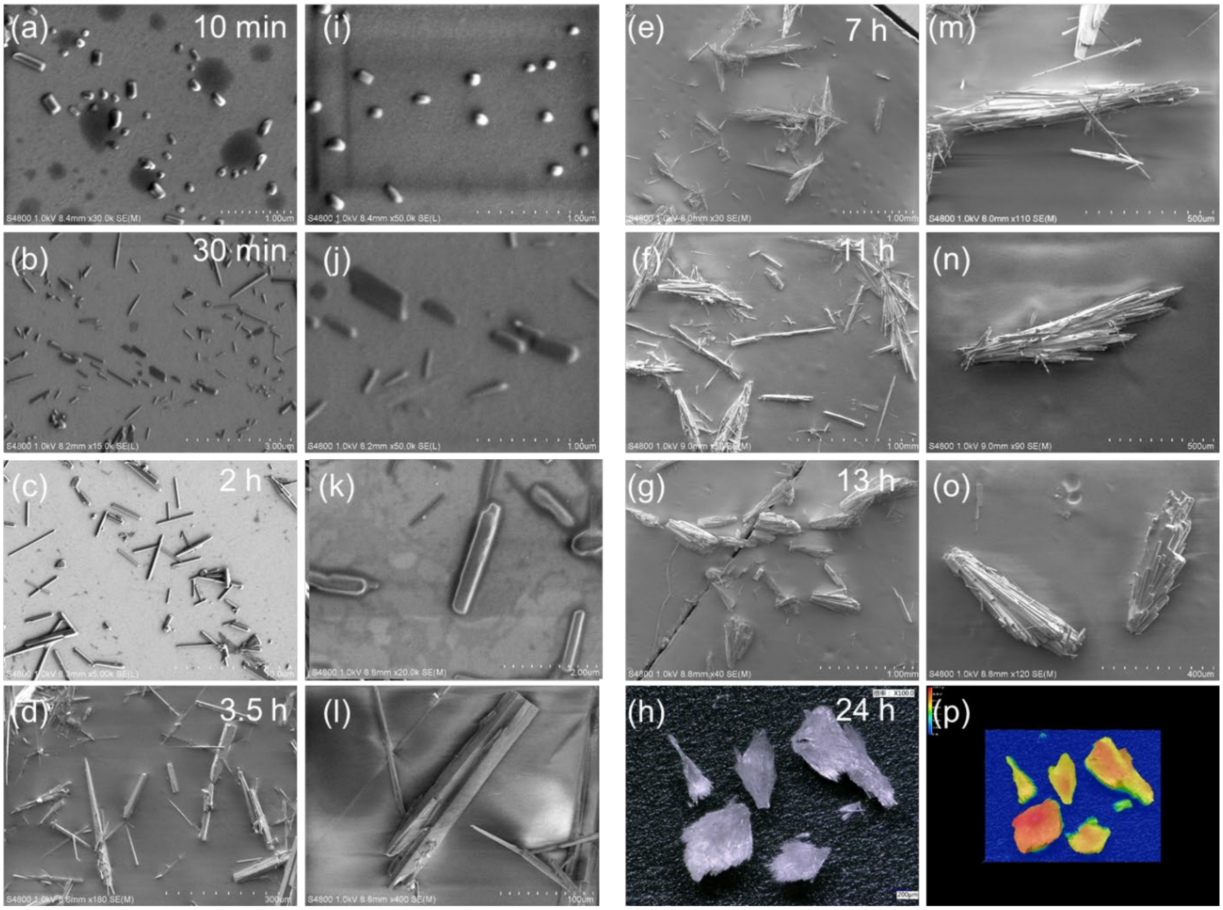

Supplementary Figure 17. The images of crystals obtained at different time when P2 was used as additive. **a-g** The SEM images of crystals obtained from 10 min to 24 h. **i-o** The enlarged SEM images corresponding to the left pictures. **h** Images obtained by optical microscope with a large depth-of-field at 24 h. **p** The height map of h.

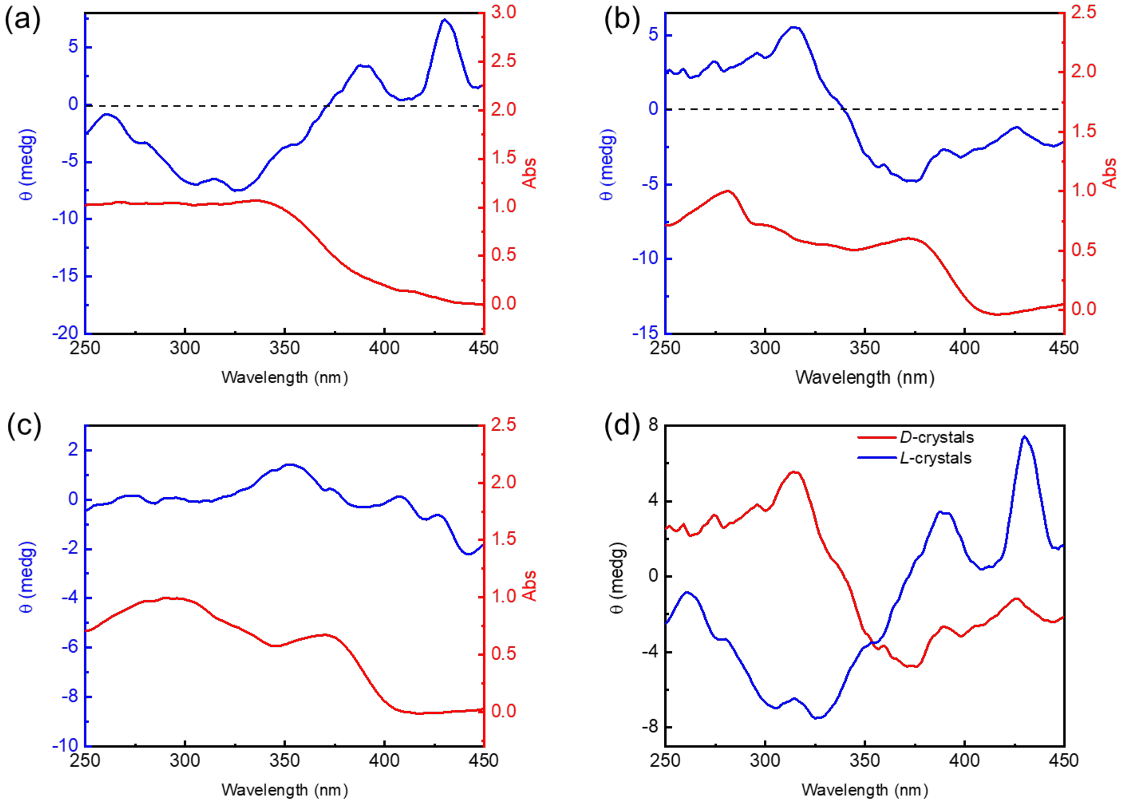

Supplementary Figure 18. The CD and adsorption spectra of the DSB labeled crystals. (a) CD (up) and adsorption (down) spectra of fan-shaped crystal aggregates of *L*-pHpgpTs when 1.5 wt% P2 was used as the additive. (b) CD (up) and adsorption (down) spectra of fan-shaped crystal aggregates of *D*-pHpgpTs when 1.5 wt% P5 was used as the additive. (c) CD (up) and adsorption (down) spectra of fan-shaped crystal aggregates of *p*Hpg*p*Ts when 1.5 wt% P9 was used as the additive.

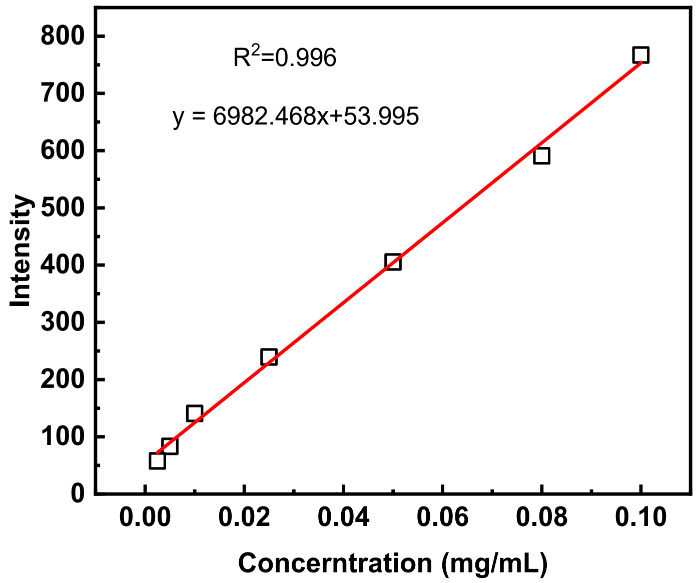

Supplementary Figure 19. Correlation between emission intensity at 515 nm and *L*-PMPA(Flu) concentration, the $R^2 = 0.996$.

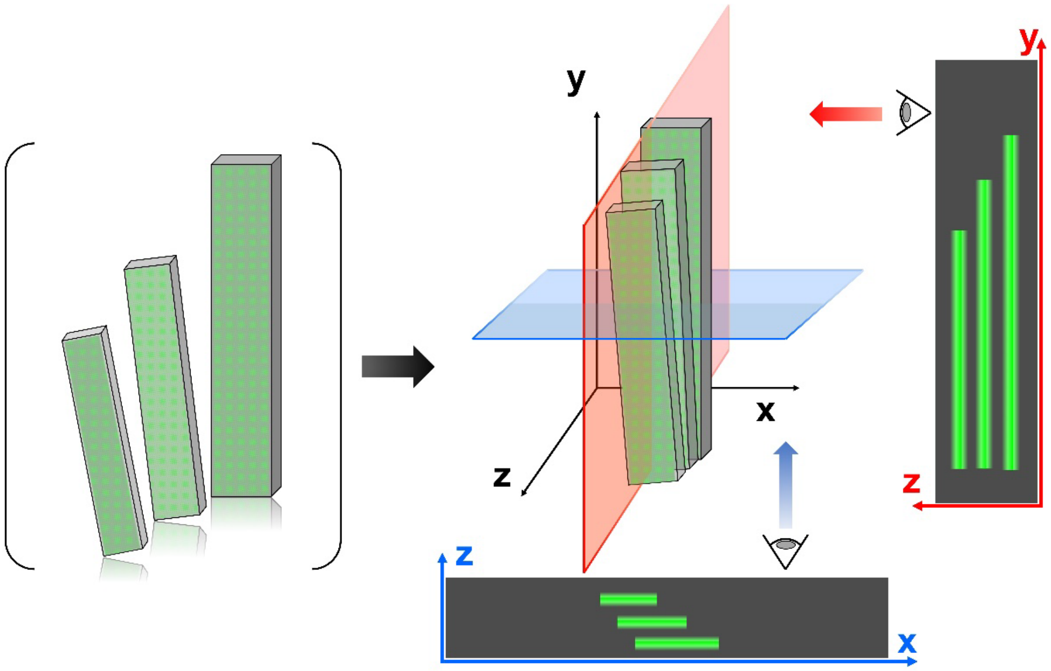

Supplementary Figure 20. The model for the situation that polymers were existed between two crystalline platelets, and the corresponding fluorescein patterns in different directions.

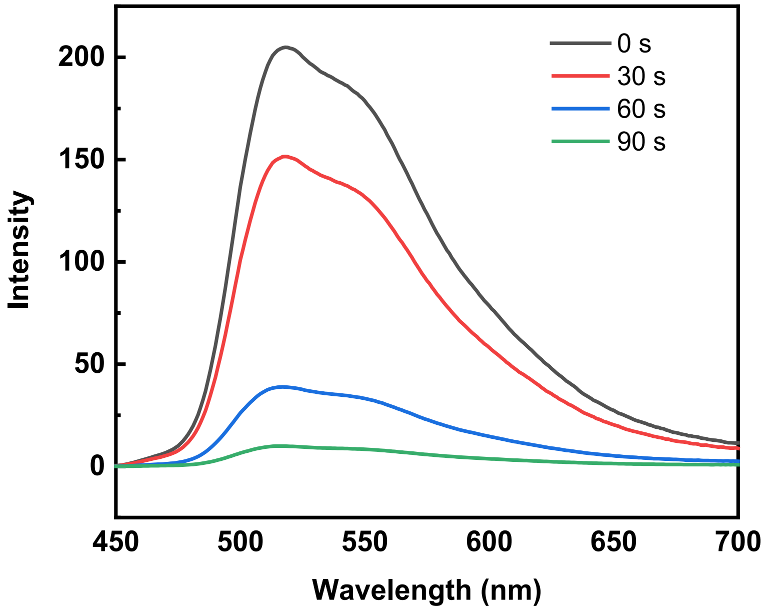

Supplementary Figure 21. Fluorescence emission spectrum of fan-shaped crystals which were treated by cold water with different time.

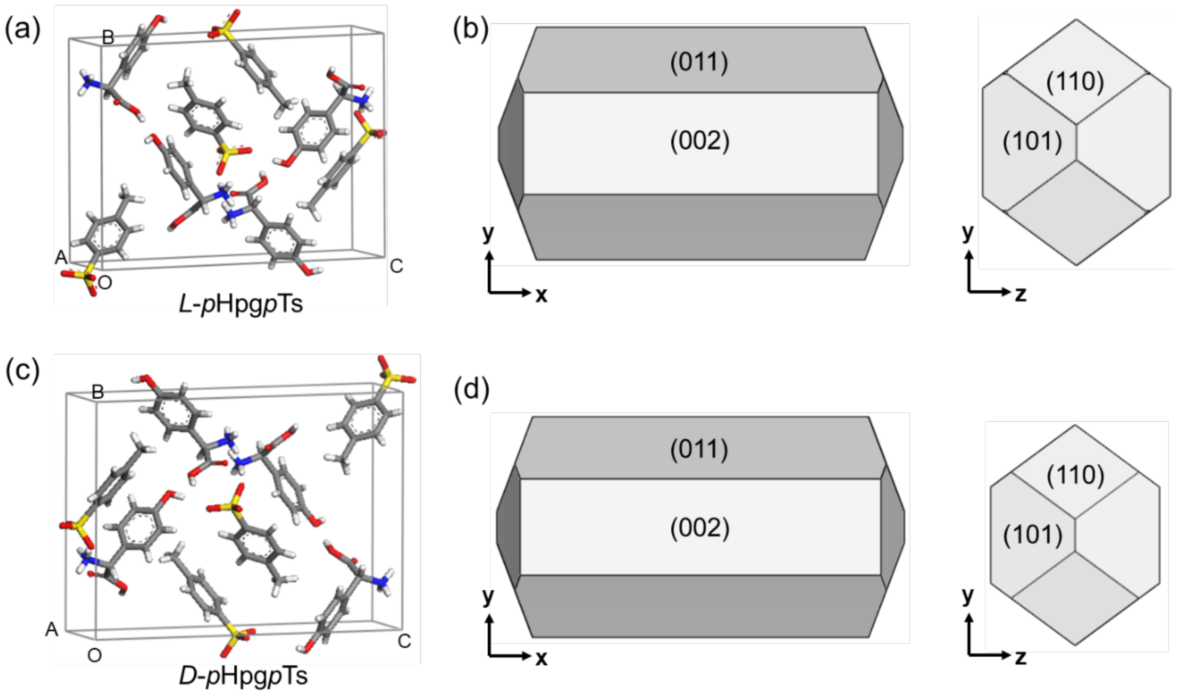

Supplementary Figure 22. Crystal structures and predicted habits in vacuum of single-crystals. **a** Crystal structure of *L*-*p*Hpg*p*Ts. **b** Predicted habit of *L*-*p*Hpg*p*Ts in vacuum. **c** Crystal structure of *D*-*p*Hpg*p*Ts. **d** Predicted habit of *D*-*p*Hpg*p*Ts in vacuum.

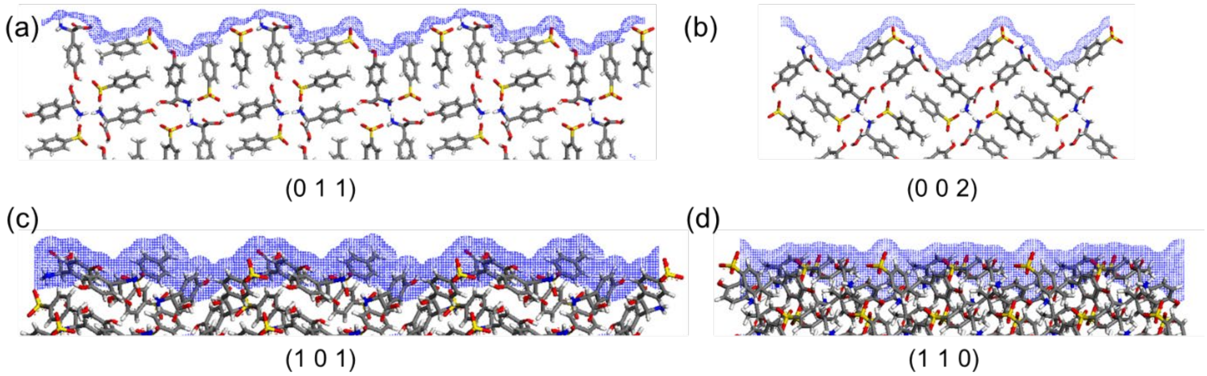

Supplementary Figure 23. The molecular arrangement of different crystal faces of *D*-*p*Hpg*p*Ts. The Connolly surfaces on the *D*-*p*Hpg*p*Ts crystal faces are denoted by the blue grid.

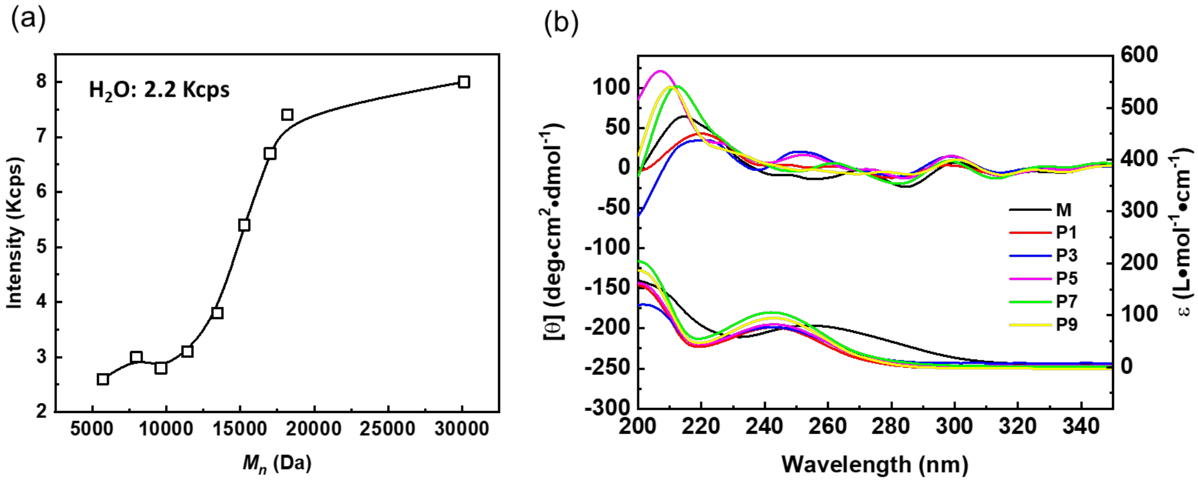

Supplementary Figure 24. Characterization of higher order chirality and possible assemblies of *L*-PMPA. **a** The correlation between scattered light intensity and M_n. The intensities of polymers (< 8Kcps) were comparable with that of water (2.2 Kcps), indicating there were no assemblies of polymers. But scattered light intensity of long chain polymers is higher than the short ones, this may be due to the slow mode of polyelectrolyte and slight collapse of polymer chains. **b** CD spectrum (up) and UV-vis spectrum (down) of the solution of the *L*-monomer and *L*-PMPA, c = 0.01 mg•mL^{-1}, solvent: water. The CD spectra of polymers were similar to the monomer's, indicating there was no higher order chiral structures.

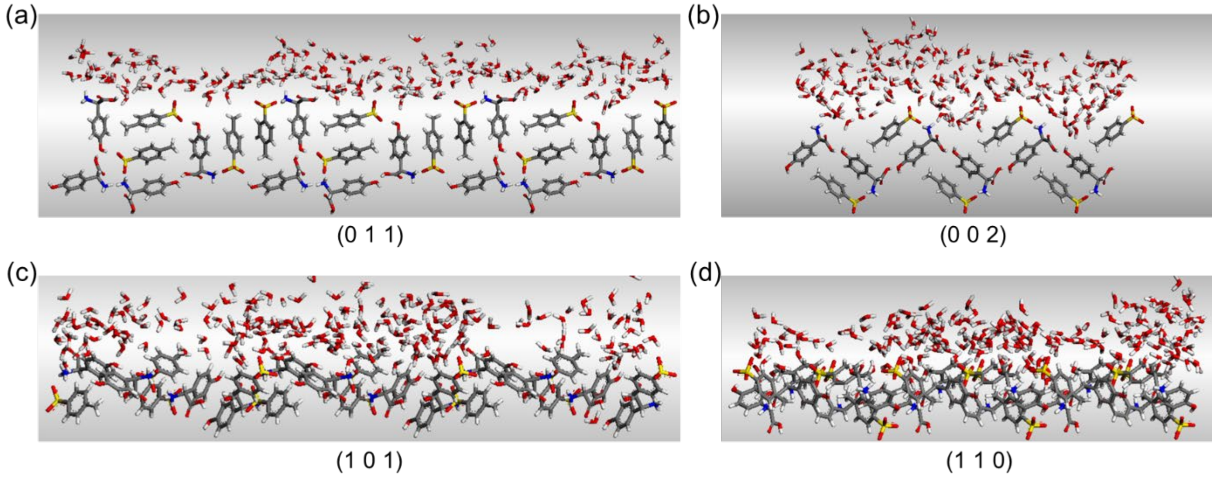

Supplementary Figure 25. Snapshots after MD simulations of the *D*-*p*Hpg*p*Ts surfaces in pure water model.

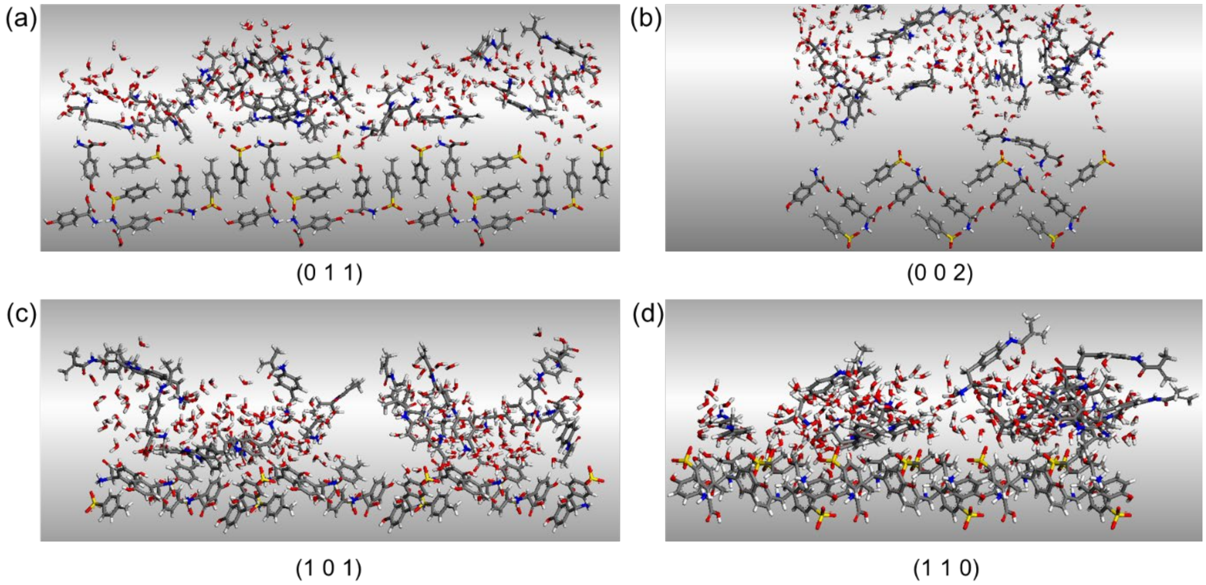

Supplementary Figure 26. Snapshots after MD simulations of the *D*-*p*Hpg*p*Ts surfaces in 7.5 mol% *L*-monomer's solution model.

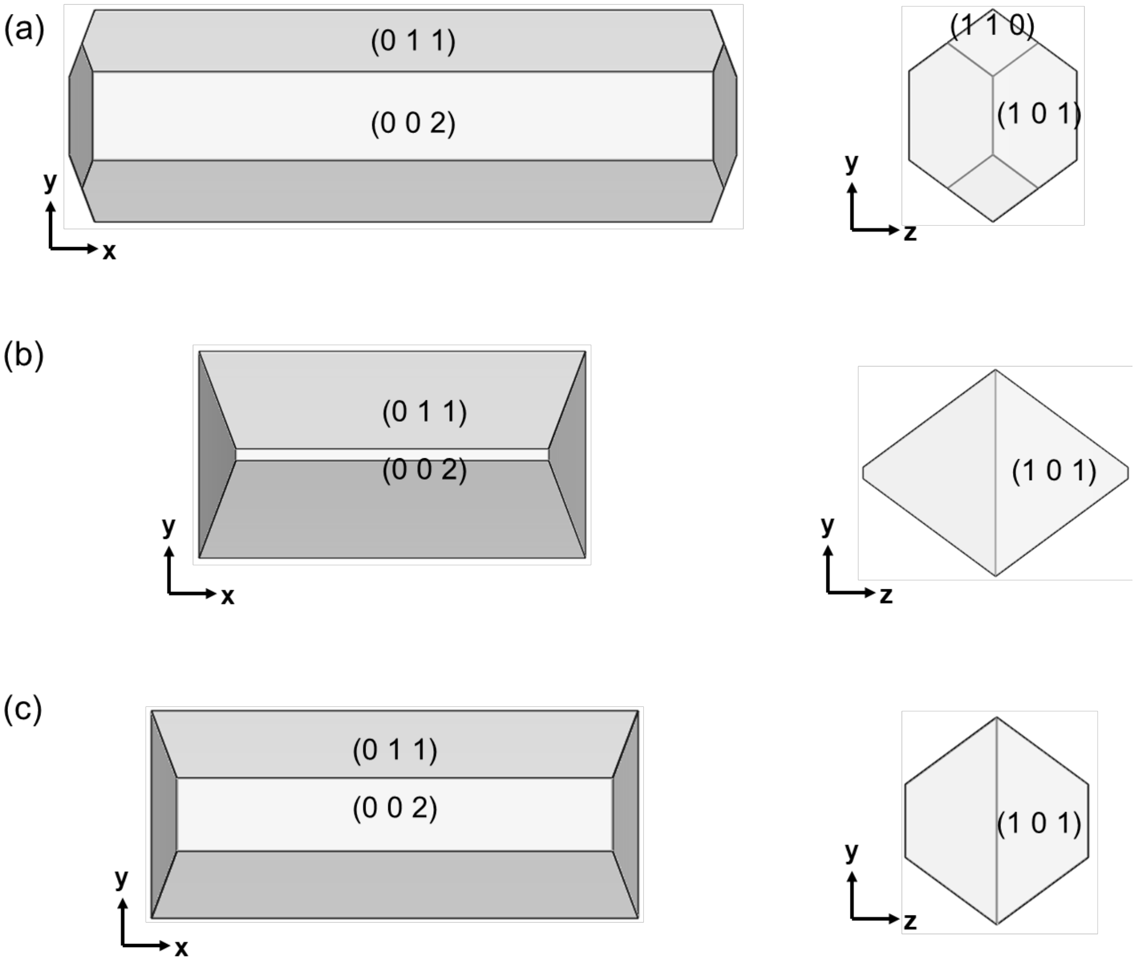

Supplementary Figure 27. The habits of *D*-*p*Hpg*p*Ts. **a** Predicted habit of *D*-*p*Hpg*p*Ts in pure water. **b** Predicted habit of *D*-*p*Hpg*p*Ts in 7.5 mol% *L*-monomer's solution. **c** Predicted habit of *D*-*p*Hpg*p*Ts in 5 mol% *L*-monomer's solution.

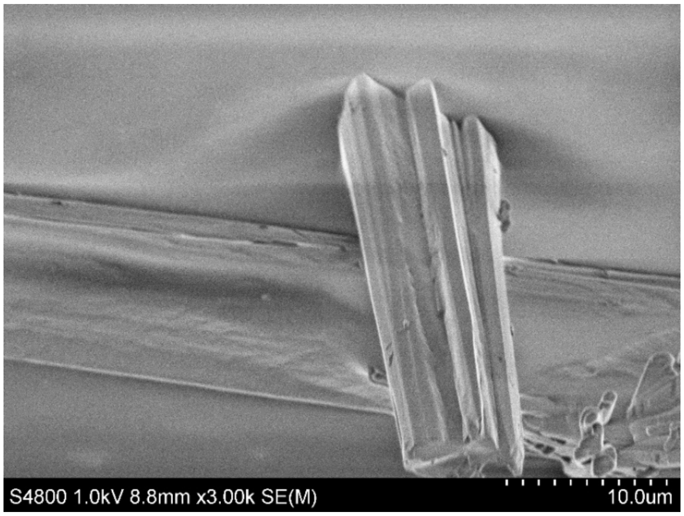

Supplementary Figure 28. The *D*-*p*Hpg*p*Ts crystals attached on the {0 1 1} faces of each other.

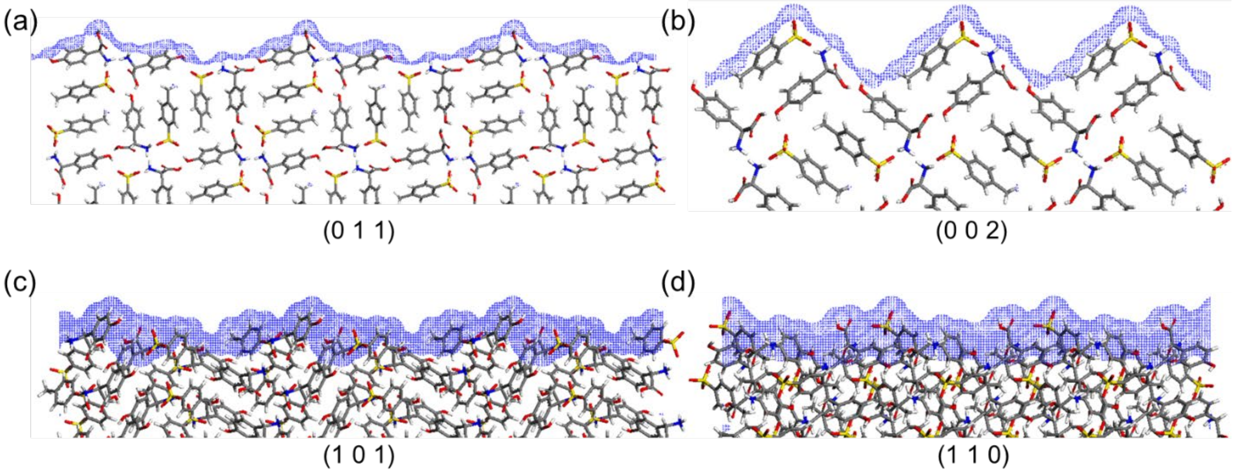

Supplementary Figure 29. The molecular arrangement of different faces of *L*-*p*Hpg*p*Ts. The Connolly surfaces on the *L*-*p*Hpg*p*Ts crystal faces are denoted by the blue grid.

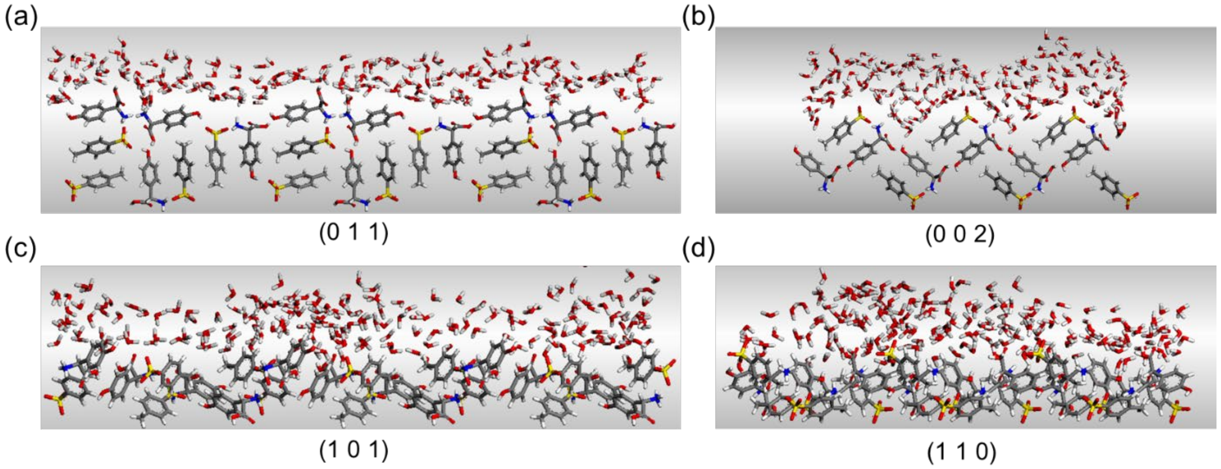

Supplementary Figure 30. Snapshots after MD simulations of the *L*-*p*Hpg*p*Ts surfaces in pure water model.

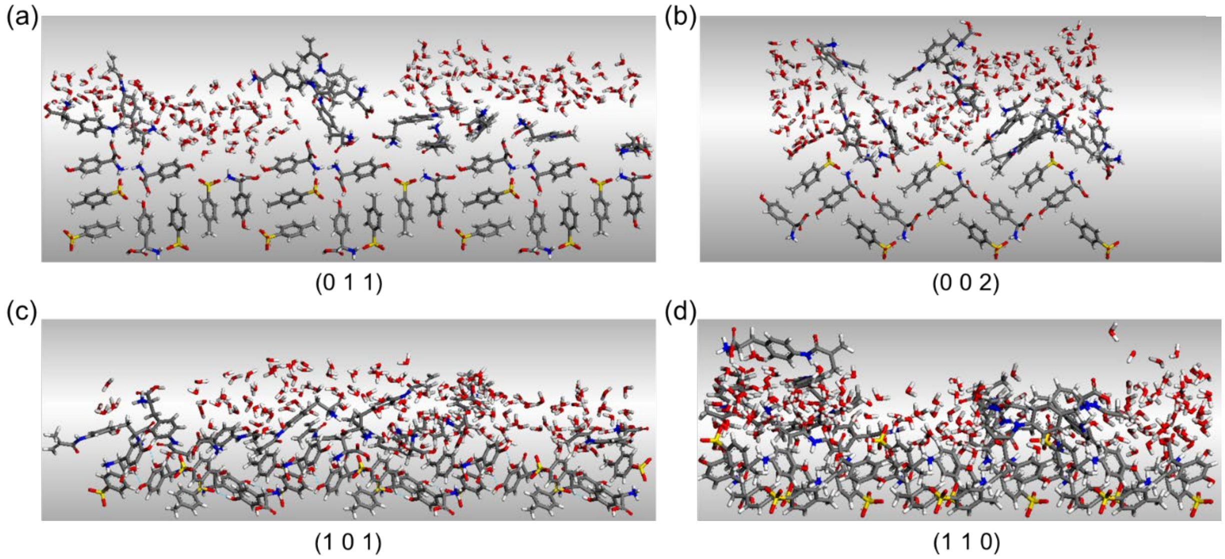

Supplementary Figure 31. Snapshots after MD simulations of the *L*-*p*Hpg*p*Ts surfaces in 5 mol% *L*-monomer's solution model.

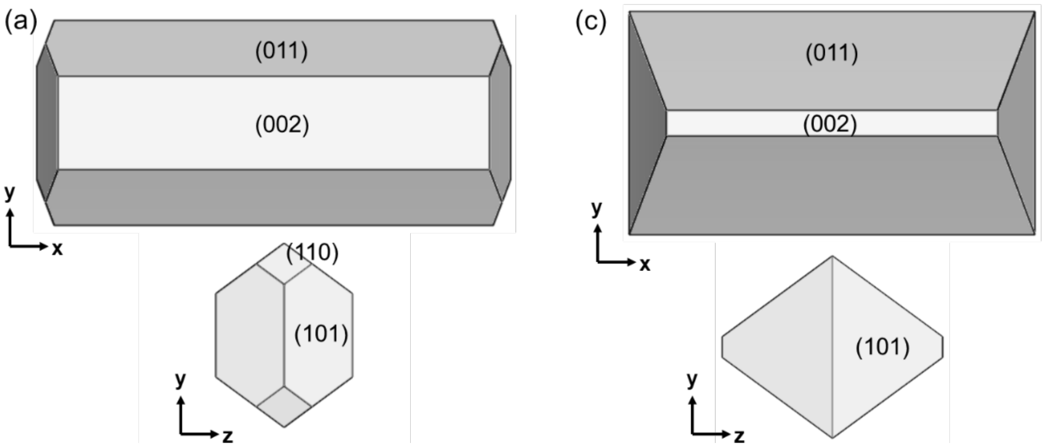

Supplementary Figure 32. The habits of single-crystals. **a** Predicted habit of *L*-*p*Hpg*p*Ts in pure water. **b** Predicted habit of *L*-*p*Hpg*p*Ts in 5 mol% *L*-monomer's solution.

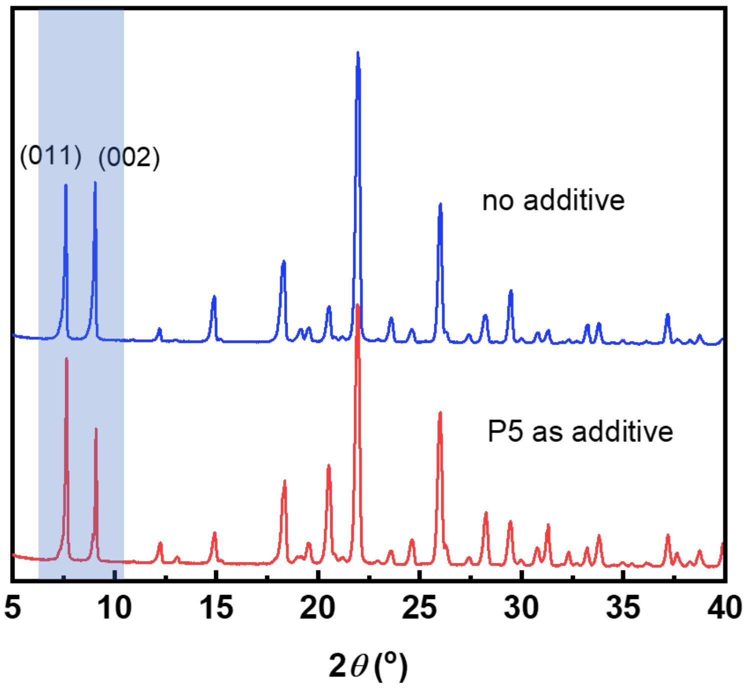

Supplementary Figure 33. Results of WAXS analysis of *D*-*p*Hpg*p*Ts crystals, blue line, default experiment in the absence of additive, red line, experiment with 1.5 $_{wt}$% of P5 was used as additive.

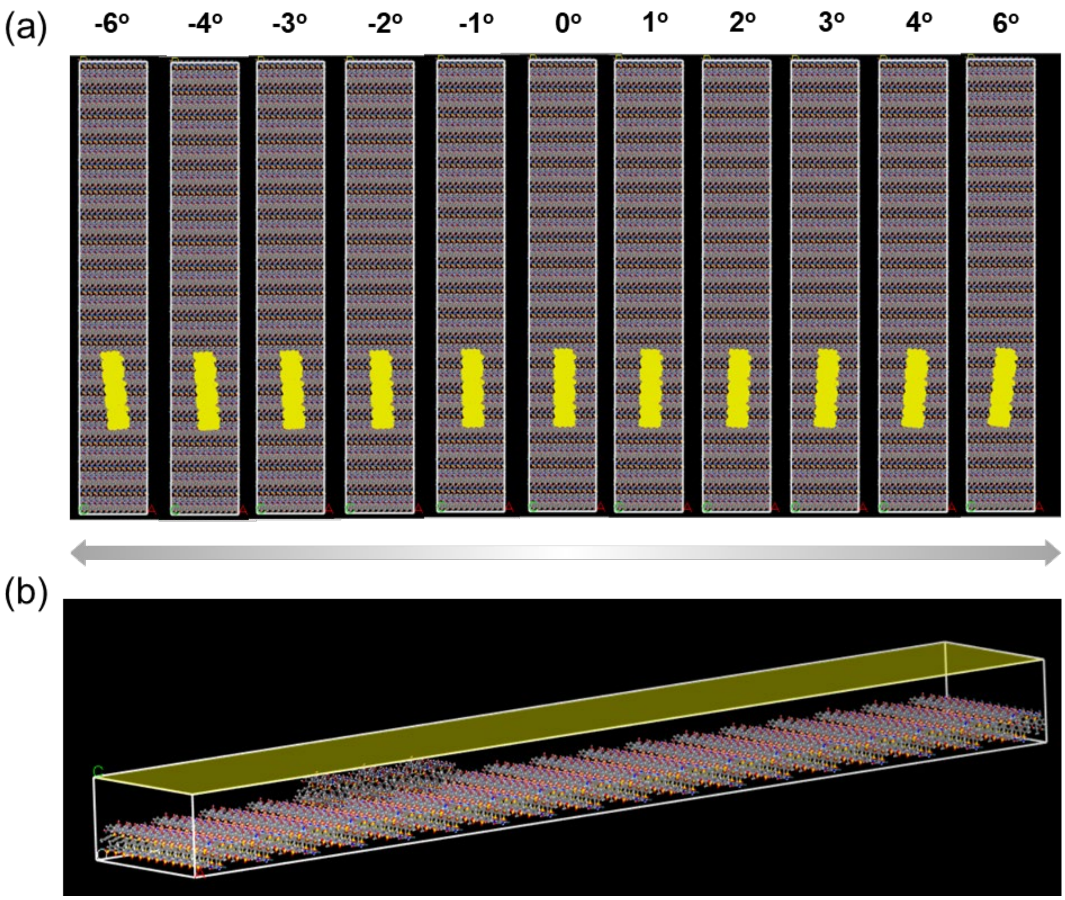

Supplementary Figure 34. The two-layered crystals' model, **a** is the top view of **b**, and the yellow parts in **a** are upper smaller crystal layers that rotate a certain angle from -6 to +6 degree.

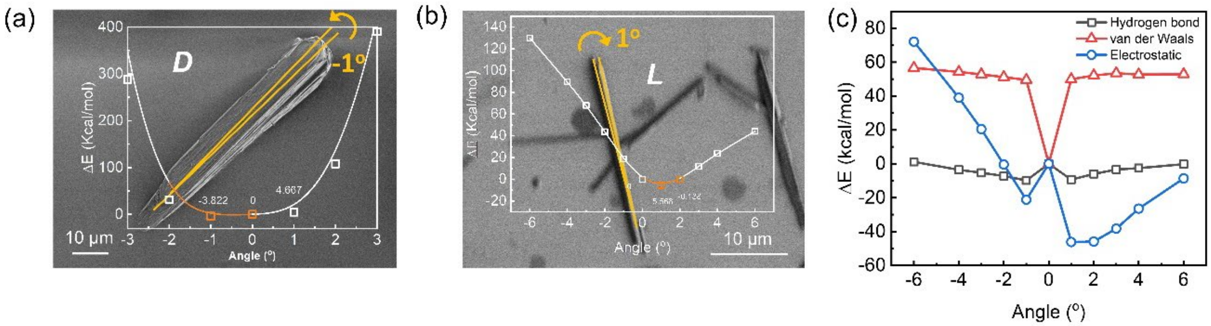

Supplementary Figure 35. Attachment angle. **a** The attachment angle (θ) of *D*-crystals (insert picture: the correlation between Δ*E* and *θ*, the total energy at 0° was defined as E_0, $\Delta E = E-E_0$). **b** The attachment angle (θ) of *L*-crystals (insert picture: the correlation between Δ*E* and *θ*). **c** The total energies were divided into three parts: hydrogen bond interactions, van der Waals forces, and electrostatic forces. The graph shows the correlation between these forces and attachment angle (θ) in *L*-crystals.

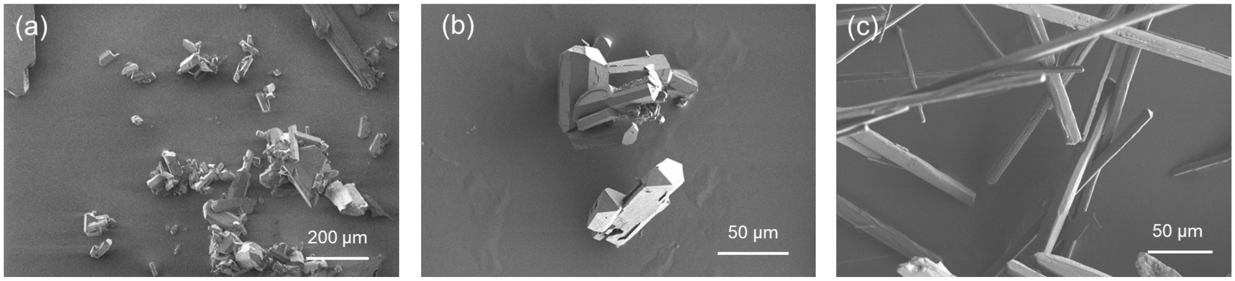

Supplementary Figure 36. Crystal morphologies of aThr. **a** The typical morphology of *D*-aThr (99.9 ee%) when 1.5 $_{wt}$% *L*-PMAL was added into the racemic solution. **b** Enlarged picture of the crystal aggregates, which were composed of several prismatic crystals. These aggregates were totally disordered. **c** The typical morphology of aThr when no additives were added in. Separated needle-like crystals can be observed.

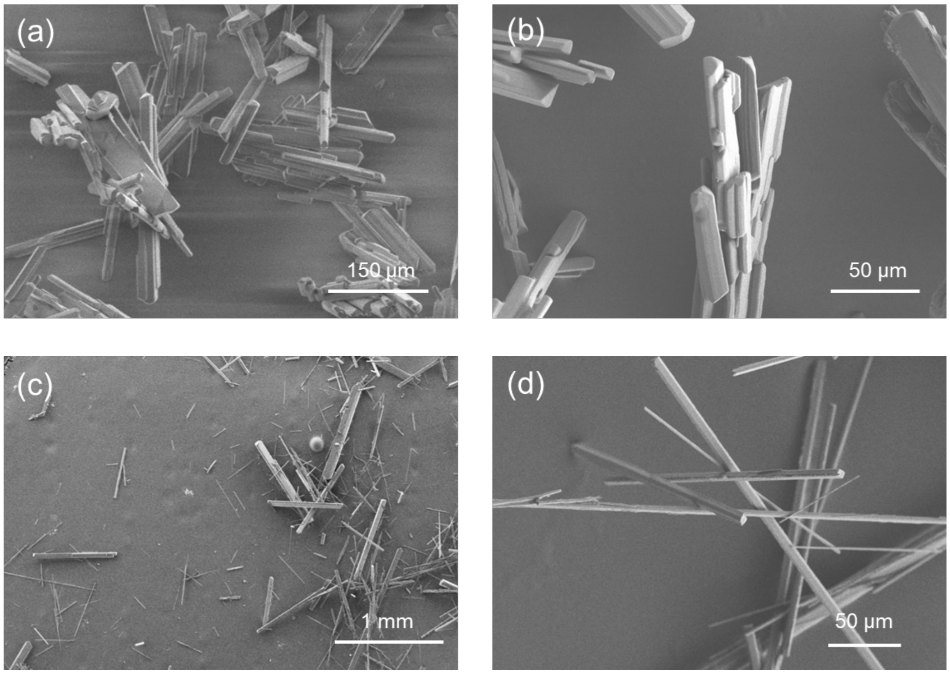

Supplementary Figure 37. Crystal morphologies of Thr. **a** The typical morphology of *D*-Thr (99.3 ee%) when 1.5 $_{\text{wt}}$% *L*-PMAL was added into the racemic solution. **b** Enlarged picture of the crystal aggregates, which were generated through oriented attachment by tiny prismatic crystals. **c-d** The typical morphology of Thr when no additives were added in. no oriented attachment has been occurred.

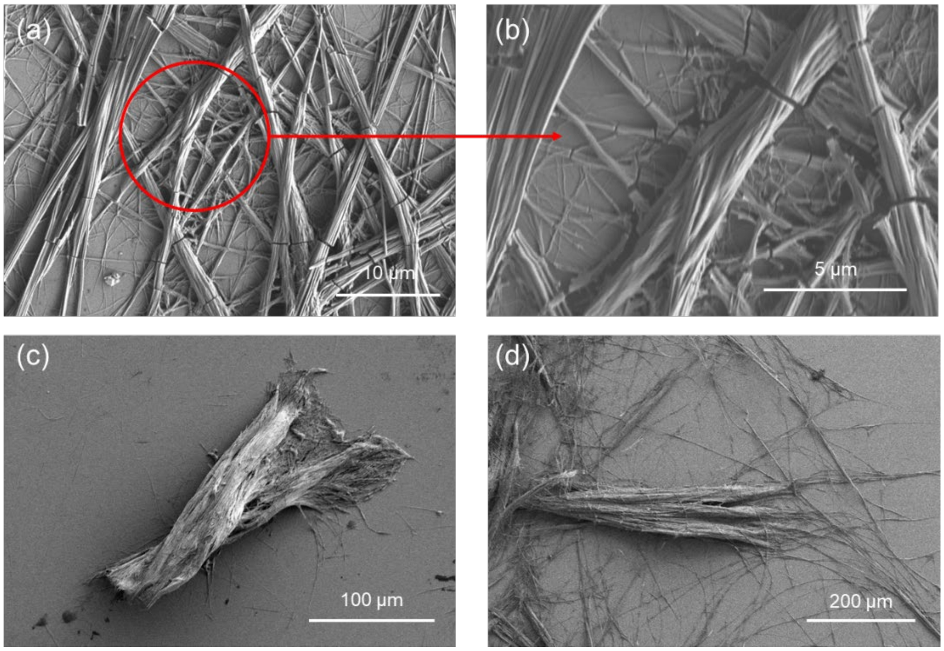

Supplementary Figure 38. Crystal morphologies of *D*-Asp_2Cu when 5 $_{wt}$% *L*-PMAL was added into the racemic solution. **a** The typical morphology of *D*-Asp_2Cu in early stage (3h). **b** Enlarged picture of the crystal aggregates. It is clear that the larger helical crystals were composed of two strands of wirelike crystals. The primary aggregates are right-handed helix while the further assembled aggregates are left-handed helix. **c-d** The typical morphology of *D*-Asp_2Cu after 6 hours of crystallization. The final helical crystal aggregates were composed of two or three strands of wirelike crystals.

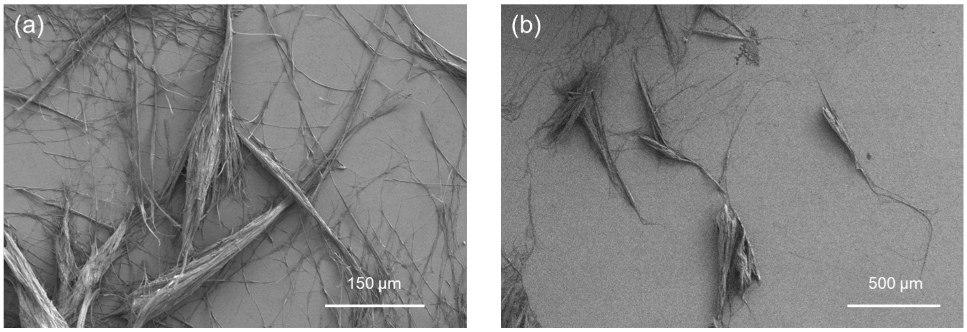

Supplementary Figure 39. Crystal morphologies of Asp_2Cu when 5 $_{wt}$% *L*-MAL was added into the racemic solution.

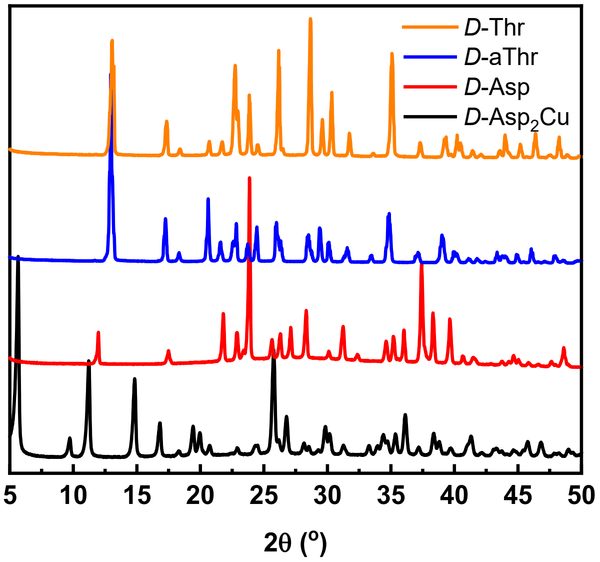

Supplementary Figure 40. WAXS analysis of the *D*-crystals with *L*-PMAL as additive, black line, *D*-Asp_2Cu; red line *D*-Asp; blue line, *D*-aThr; orange line, *D*-Thr. The shape diffraction peaks indicated that these obtained solids were crystals rather than gels or supramolecular assemblies.

Supplementary Table 1. RAFT polymerization results of *L*-PMPABoc[a]

Polymer	Retention time	M_n [b]	M_w	PDI
P1-Boc	23.798	5700	6200	1.05
P2-Boc	22.966	8000	8400	1.05
P3-Boc	22.453	9600	10500	1.08
P4-Boc	22.073	11400	12600	1.06
P5-Boc	21.653	13500	15000	1.10
P6-Boc	21.303	15300	17500	1.11
P7-Boc	21.203	17000	18800	1.10
P8-Boc	21.073	18200	20200	1.11
P9-Boc	19.739	30100	35800	1.18
L-PMPA(Flu)-Boc	21.455	14400	18100	1.26

[a] Monomer concentration = 0.16 g mL^{-1}; temperature, 80 ºC; solvent, dioxane. [b] Obtained from the corresponding $PMPABocOCH_3$.

Supplementary Table 2. The morphologies and chirality of crystals obtained by using polymeric additives with various molar masses

	**P1-P3**		**P4-P6**	**P7-P9**
**Morphologies**	Needle-like (minor) + Fan-shaped (major)		Fan-shaped	Fan-shaped
**Crystal chirality**	*D*-crystals	*L*-crystals dominated	*D*-crystals dominated	*D*- and *L*-crystals
**Overall chirality**	*L*-*p*Hpg*p*Ts dominated		*D*-*p*Hpg*p*Ts dominated	Racemic

Supplementary Table 3. Crystal habit parameters of *L*-*p*Hpg*p*Ts in vacuum predicted by the AE model

Faces	d/Å[a]	E_{att} (kcal/mol)	R[b]	Total facet area (%)[c]
{0 1 1}	11.60	-41.99	1	53.41
{0 0 2}	9.78	-39.81	0.95	24.98
{1 0 1}	5.25	-87.23	2.08	11.57
{1 1 0}	5.10	-87.29	2.08	10.02

[a] The interplanar distance, not the distance of the crystal surface to the center (D(hkl)); [b] Relative growth rate in vacuum, taking the growth rate of {0 1 1} as 1; [c] The ratio of the {h k l} surfaces area to the total surface area.

Supplementary Table 4. Crystal habit parameters of *D*-*p*Hpg*p*Ts in vacuum predicted by the AE model

Faces	d/Å[a]	E_{att} (kcal/mol)	R[b]	Total facet area (%)[c]
{0 1 1}	11.60	-32.39	1	54.36
{0 0 2}	9.78	-30.81	0.95	25.18
{1 0 1}	5.25	-71.55	2.21	11.05
{1 1 0}	5.10	-71.51	2.21	9.40

[a] The interplanar distance, not the distance of the crystal surface to the center (D(hkl)); [b] Relative growth rate in vacuum, taking the growth rate of {0 1 1} as 1; [c] The ratio of the {h k l} surfaces area to the total surface area.

Supplementary Table 5. Calculated parameters for dominant faces of *L*-*p*Hpg*p*Ts in pure water.

Faces	E_{int}[a] (kcal/mol)	A_{acc}[b] (Å^2)	A_{model} (Å^2)	E_s[c] (kcal/mol)	E_{att}'[d] (kcal/mol)	R'[e]	Total facet area (%)[f]
{0 1 1}	-108.28	1599.36	1191.30	-16.15	-25.84	1	56.92
{0 0 2}	-93.57	1085.30	706.06	-15.98	-23.82	0.92	27.10
{1 0 1}	-93.64	4009.20	2631.82	-15.85	-71.38	2.76	13.92
{1 1 0}	-68.62	4162.93	2710.83	-11.71	-75.58	2.92	2.06

[a] Obtained by conducting dynamic simulation, see details in supplementary methods; [b] Obtained by calculating the Connolly surfaces; [c] $E_s = (E_{int}*A_{acc})/(9*A_{model})$; [d] $E_{att}' = E_{att}-E_s$; [e] Relative growth rate in water, taking the growth rate of {0 1 1} as 1; [f] The ratio of the {h k l} surfaces area to the total surface area.

Supplementary Table 6. Calculated parameters for dominant faces of *L*-*p*Hpg*p*Ts in *L*-monomer's aqueous solution (5 mol%)[a].

Faces	E_{int}[b] (kcal/mol)	A_{acc}[c] (Å^2)	A_{model} (Å^2)	E_s[d] (kcal/mol)	E_{att}'[e] (kcal/mol)	R'[f]	Total facet area (%)[g]
{0 1 1}	-130.06	1599.36	1191.30	-19.40	-22.59	1	74.09
{0 0 2}	-35.19	1085.30	706.06	-6.01	-33.80	1.50	5.03
{1 0 1}	-225.04	4009.20	2631.82	-38.10	-49.14	2.18	20.88
{1 1 0}	83.95	4162.93	2710.83	14.32	-101.61	4.50	0

[a] Obtained by conducting dynamic simulation, see details in supplementary methods; [b] Obtained by calculating the Connolly surfaces; [c] $E_s = (E_{int}*A_{acc})/(9*A_{model})$; [d] $E_{att}' = E_{att}-E_s$; [e] Relative growth rate in *L*-monomer's aqueous solution, taking the growth rate of {0 1 1} as 1; [f] The ratio of the {h k l} surfaces area to the total surface area.

Supplementary Table 7. Calculated parameters for dominant faces of *D-p*Hpg*p*Ts in pure water.

Faces	E_{int}[a] (kcal/mol)	A_{acc}[b] (Å^2)	A_{model} (Å^2)	E_s[c] (kcal/mol)	E_{att}'[d] (kcal/mol)	R'[e]	Total facet area (%)[f]
{0 1 1}	-110.46	1663.06	1192.89	-17.11	-15.28	1	61.82
{0 0 2}	-92.27	1088.32	707.12	-15.78	-15.03	0.98	25.80
{1 0 1}	-84.80	4004.86	2714.72	-13.90	-57.61	3.77	9.33
{1 1 0}	-73.90	4216.28	2635.67	-13.14	-58.41	3.82	3.06

[a] Obtained by conducting dynamic simulation, see details in supplementary methods; [b] Obtained by calculating the Connolly surfaces; [c] $E_s = (E_{int}*A_{acc})/(9*A_{model})$; [d] $E_{att}' = E_{att}-E_s$; [e] Relative growth rate in water, taking the growth rate of {0 1 1} as 1; [f] The ratio of the {h k l} surfaces area to the total surface area.

Supplementary Table 8. Calculated parameters for dominant faces of *D-p*Hpg*p*Ts in *L*-monomer's aqueous solution (5 mol%)[a].

Faces	E_{int}[b] (kcal/mol)	A_{acc}[c] (Å^2)	A_{model} (Å^2)	E_s[d] (kcal/mol)	E_{att}'[e] (kcal/mol)	R'[f]	Total facet area (%)[g]
{0 1 1}	-120.46	1663.06	1192.89	-18.66	-13.73	1	64.24
{0 0 2}	-92.27	1088.32	707.12	-15.78	-15.03	1.09	19.46
{1 0 1}	-200.90	4004.86	2714.72	-32.93	-38.58	2.81	16.29
{1 1 0}	14.80	4216.28	2635.67	2.63	-74.18	5.40	0

[a] In 200 water molecules, 10 molecules were replaced by *L*-monomer. [b] Obtained by conducting dynamic simulation, see details in supplementary methods; [c] Obtained by calculating the Connolly surfaces; [d] $E_s = (E_{int}*A_{acc})/(9*A_{model})$; [e] $E_{att}' = E_{att}-E_s$; [f] Relative growth rate in *L*-monomer's aqueous solution, taking the growth rate of {0 1 1} as 1; [g] The ratio of the {h k l} surfaces area to the total surface area.

Supplementary Table 9. Calculated parameters for dominant faces of *D*-*p*Hpg*p*Ts in *L*-monomer's aqueous solution (7.5 mol%)[a].

Faces	E_{int}[a] (kcal/mol)	A_{acc}[b] (Å^2)	A_{model} (Å^2)	E_s[c] (kcal/mol)	E_{att}'[d] (kcal/mol)	R'[e]	Total facet area (%)[f]
{0 1 1}	-126.54	1663.06	1192.89	-19.60	-12.79	1	77.51
{0 0 2}	-60.85	1088.32	707.12	-10.41	-20.40	1.59	2.36
{1 0 1}	-261.20	4004.86	2714.72	-42.81	-28.70	2.24	20.13
{1 1 0}	19.38	4216.28	2635.67	3.44	-74.99	5.86	0

[a] In 200 water molecules, 15 molecules were replaced by *L*-monomer. [b] Obtained by conducting dynamic simulation, see details in supplementary methods; [c] Obtained by calculating the Connolly surfaces; [d] $E_s = (E_{int}*A_{acc})/(9*A_{model})$; [e] $E_{att}' = E_{att}-E_s$; [f] Relative growth rate in *L*-monomer's aqueous solution, taking the growth rate of {0 1 1} as 1; [g] The ratio of the {h k l} surfaces area to the total surface area.

Supplementary References:

1 Ning, Y. et al. What dictates the spatial distribution of nanoparticles within calcite? *J. Am. Chem. Soc.* **141**, 2481-2489 (2019).

2 Hartman, P. & Perdok, W. G. On the relations between structure and morphology of crystals .2. *Acta Crystallographica* **8**, 521-524 (1955).

3 Hartman, P. & Bennema, P. The attachment energy as a habit controlling factor .1. Theoretical considerations. *J. Cryst. Growth* **49**, 145-156 (1980).

4 Wang, C. et al. Effects of solvent and supersaturation on crystal morphology of cefaclor dihydrate: a combined experimental and computer simulation study. *CrystEngComm* **18**, 9085-9094 (2016).

5 Duan, X. H. et al. A molecular dynamics simulation of solvent effects on the crystal morphology of HMX. *J. Hazard Mater.* **174**, 175-180 (2010).

6 Schmidt, C. & Ulrich, J. Predicting crystal morphology grown from solution. *Chem. Eng. Technol.* **35**, 1009–1012 (2012).

7 Liang, Z. Z. et al. Qualitative rationalization of the crystal growth morphology of benzoic acid controlled using solvents. *CrystEngComm* **16**, 5997–6002 (2014).
